# Copper‐Zinc Bimetallic Two‐Dimensional Conjugated Coordination Polymers for Highly‐Selective Electrochemical CO_2_ Reduction to Ethanol

**DOI:** 10.1002/smll.73914

**Published:** 2026-05-26

**Authors:** Rashid Iqbal, Tianchun Li, Zhao Yan, Geping Zhang, Fengxiang Zhao, Huan Huang, Hua Wang, Yu Jing, Jingcheng Hao, Renhao Dong

**Affiliations:** ^1^ Department of Chemistry The University of Hong Kong Hong Kong China; ^2^ Materials Innovation Institute for Life Sciences and Energy (MILES) HKU‐SIRI Shenzhen China; ^3^ Jiangsu Co‐Innovation Centre of Efficient Processing and Utilization of Forest Resources, College of Chemical Engineering Nanjing Forestry University Nanjing China; ^4^ Institute of High Energy Physics Chinese Academy of Sciences Beijing China; ^5^ Key Laboratory of Colloid and Interface Chemistry of the Ministry of Education, School of Chemistry and Chemical Engineering Shandong University Jinan China

**Keywords:** 2D coordination polymers, bimetallic active sites, carbon dioxide reduction reaction, electrocatalysis, electrocatalysts

## Abstract

Copper‐based catalysts are the most promising catalysts for their ability to electrochemically prepare multi‐electron C_2+_ products like ethanol (C_2_H_5_OH). However, the challenges persist in achieving high yields and selectivity, attributed to the unstable intermediates during the CO_2_ reduction reaction (CO_2_RR). Here, we report layer‐stacked two‐dimensional conjugated coordination polymers (2D c‐CPs) with spatially separated Cu/Zn‐S_4_ bi‐active sites (named as BHT‐Cu_x_‐Zn_y_, x + y = 1) as electrocatalysts for addressing the above challenges of low Faradaic efficiency (FE) and subpar selectivity for ethanol production. Notably, the resultant BHT‐Cu_0.8_‐Zn_0.2_ 2D c‐CP exhibits boosting electrochemical selective conversion of CO_2_ to C_2_H_5_OH with a remarkable FE of 92.3 ± 2.4% at 126.7 mA cm^−2^ in a flow‐cell, with stability maintained over 150 h, superior to the thus‐far‐reported Cu‐based electrocatalysts. The integration of Zn enhances the catalytic activity and the interaction strength of Cu with key intermediate compounds. Benefited from the high selectivity, we further present a carbon efficiency of 73% for the conversion of CO_2_ to C_2_H_5_OH, along with a full‐cell energy efficiency of 52%. Additionally, the energy cost is calculated at 56.6 GJ per tonne of C_2_H_5_OH, which is the lowest reported among the existing CO_2_ electrolysis systems for C_2_H_5_OH production.

## Graphical abstract

Two‐dimensional conjugated coordination polymers featuring spatially separated Cu/Zn–S_4_ bi‐active sites enable the selective electrochemical conversion of CO_2_ to ethanol via stabilized C_2_ intermediates, achieving a Faradaic efficiency of 92.3% at a current density exceeding 120 mA cm^−^^2^ and an energy cost of 56.6 GJ per ton.

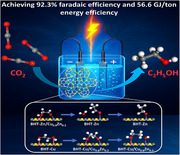

## 1 Introduction

**FIGURE 1:**
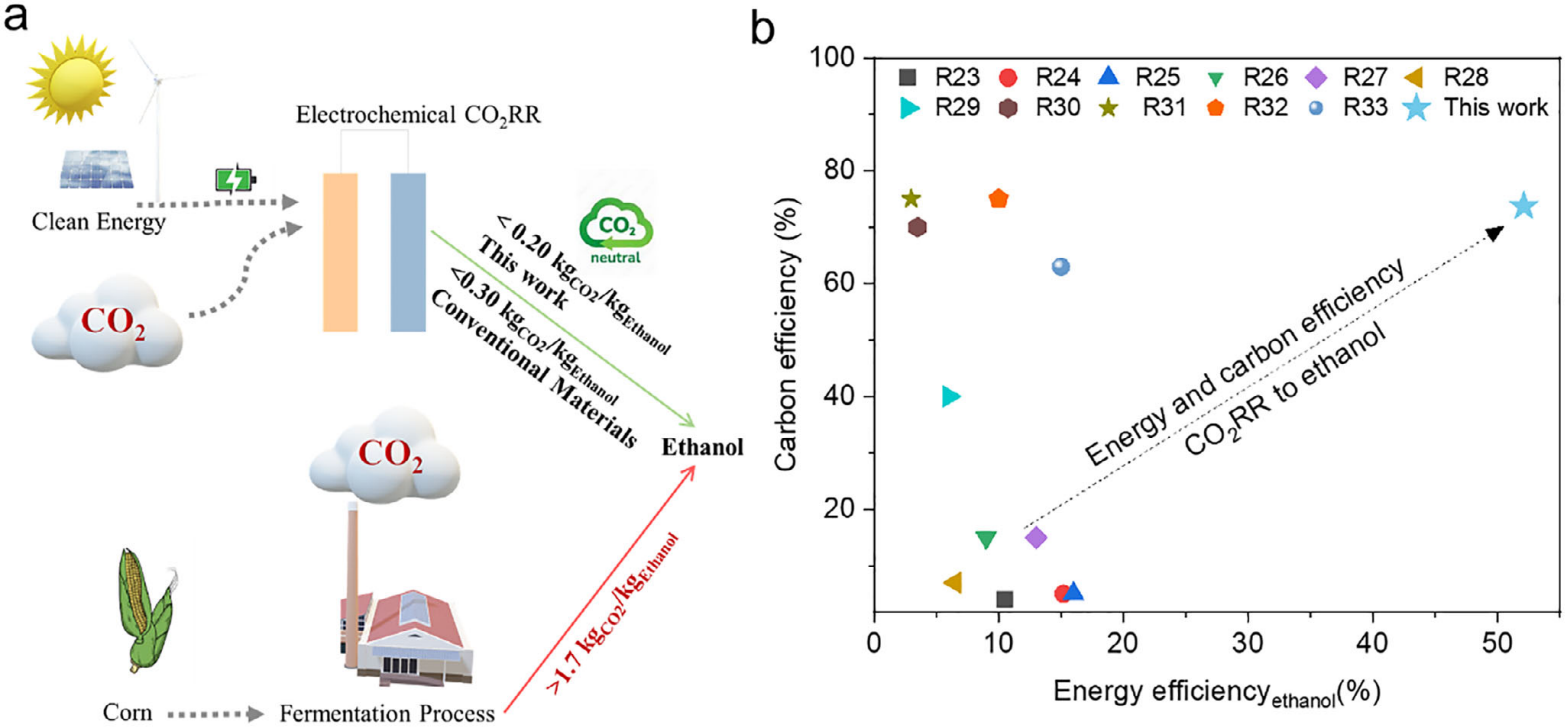
Evaluation of carbon intensity and performance of CO_2_RR in electrolyzers. (a) The carbon intensity associated with C_2_H_5_OH production from both CO_2_RR and biomass sources [14]. The carbon intensity for the CO_2_RR process was derived based on the carbon intensity of the renewable electricity utilized by the electrolyzer. (b) A comparative analysis of carbon and energy efficiency for C_2_H_5_OH electrosynthesis across benchmark CO_2_ electrolyzers [15, 16, 17, 18, 19, 20, 21, 22, 23, 24, 25].

Ethanol is important for various industries due to its versatile applications and potential as a renewable fuel source. It primarily serves as a solvent in pharmaceuticals, alcoholic beverages, and as a feedstock for chemicals, plastics, and biofuel additives in gasoline [1, 2, 3]. The key determinants of ethanol production levels and cost are its consumption patterns and the overall market demand [4]. In 2024, the global ethanol production was approximately 122 billion liters, with the United States, Brazil, and the European Union being the leading producers [2, 4, 5]. The cost of ethanol production depends on factors such as feedstock prices, energy costs, and production efficiency, but it typically ranges from $0.40 to $1.00 per liter [2]. Although biomass‐based ethanol production has become a crucial platform, its carbon intensity remains considerably high, exceeding 1.7 kg_CO2_/kg_Ethanol_ (Figure 1a). Thus, decarbonizing ethanol production is a vital objective for reducing CO_2_ emissions and dropping the production cost of ethanol. Therefore, the electrochemical conversion of carbon dioxide into ethanol can help address challenges associated with waste CO_2_ disposal through lower carbon intensity of <0.30 kg_CO2_/kg_Ethanol_ and contribute to the development of sustainable carbon utilization technologies. According to techno‐economic analysis, the higher current density of 100 mA cm^−2^, lower overpotential than 0.5 V, and above 90% Faradaic efficiency (FE) are three basic parameters that can make the electrochemical CO_2_RR industrially feasible and profitable [6].

Copper demonstrates a highly efficient electrocatalyst toward CO_2_RR and shows a remarkable capacity to generate various compounds beyond CO*, such as alcohols and hydrocarbons [7]. Among Cu‐based catalysts, Cu_2_O is the state‐of‐the‐art electrocatalyst for ethanol formation but is limited to the low selectivity of around 60% for ethanol, the competing reaction kinetics, the surface poisoning, and the stability issues, which have hindered its practical application [8, 9]. As an alternative, Cu–Zn bimetallic alloys have garnered interests recently due to the low cost, while Zn is considerably more common and far less expensive than Cu. Due to the poor activity of Zn toward the hydrogen evolution reaction (HER), the addition of Zn to the Cu electrode is anticipated to prevent the production of H_2_ [10]. Furthermore, the fine‐tuning of the Zn content in Cu–Zn bimetallic catalysts can vary the selectivity for C_2_H_5_OH as opposed to ethylene (C_2_H_4_). For instance, bimetallic oxide‐derived Cu_x_Zn catalysts with varying Zn content show that the Cu_4_Zn_1_ catalyst achieved a peak C_2_H_5_OH FE of 29.1%. In contrast, the Cu_2_Zn_1_ catalyst exhibited a maximum ethanol‐to‐ethylene FE ratio of 6, with an ethanol FE of 24% and an ethylene FE of 4%. Despite the significant potential of Cu–Zn alloys for CO_2_‐to‐C_2_H_5_OH conversion, they have a limited electrochemically active surface area for CO_2_RR due to their bulk size. The scarcity of well‐distributed Cu/Zn interfaces due to their disordered arrangement within the alloys usually impeded the efficient utilization of active sites, leading to a carbon product mixture rather than selective C_2_H_5_OH production [11, 12]. Thus, the C_2_H_5_OH produced in Cu–Zn alloys is inadequate; the FE (<50%) is not satisfying the manufacturing demands [13], which highlights the urgent requirement for the development of a robust electrocatalyst for gaining high and stable FE of C_2_H_5_OH during CO_2_RR.

Crystalline coordination polymers or metal‐organic frameworks (MOFs) present distinct advantages compared with single‐atom catalysts (SACs) due to their well‐defined porous structures [26, 27], tuneable and spatially arranged active sites [28], and versatility [29, 30]. With MOFs, the precise arrangement of metal ions and organic ligands offers a controlled environment for catalysis, facilitating mechanistic studies and enabling high performance in electroreduction reactions [31]. Despite increasing interests in MOF electrocatalysts, only a few reports have evaluated their electrocatalytic ability to convert CO_2_ into added‐value compounds. Nevertheless, inspired by the design of Cu–Zn alloys for converting CO_2_ to C_2_H_5_OH, Cu–Zn bimetallic MOFs have been emerging for electrochemical CO_2_RR, which unfortunately only generated CO+H_2_ syngas [32]. Recent studies have revealed that the electrochemical CO_2_RR for C_2_H_5_OH using Cu–Zn heterometallic MOFs showed sluggish stability with FE of 25%. Specifically, the electrocatalyst based on the ubiquitous microporous HKUST‐1 displayed the record results in terms of reaction rates and FE of 47.2% toward the overall production of C_2_H_5_OH and CH_3_OH among the evaluated MOFs [33], but was still unable to compete with numerous state‐of‐the‐art Cu‐based electrocatalysts.

Here, we present a sacrificial template‐assisted bimetallic synthesis (STAB) strategy to yield spatially separated Cu‐/Zn‐S_4_ bi‐active sites for addressing challenges in copper‐based catalysts for CO_2_ electroreduction. This STAB strategy utilizes traditional three‐dimensional HKUST‐1 MOF as sacrificial templates to synthesize conductive BHT‐Cu_x_‐Zn_y_ **(x + y = 1)** 2D conjugated coordination polymers (2D c‐CPs). Employing Cu/Zn bimetals in various ratios linked with benzene hexathiol (BHT) ligand, the resultant BHT‐Cu_0.8_‐Zn_0.2_ exhibits boosting selective conversion of CO_2_ to C_2_H_5_OH with a remarkable FE of 93.4 ± 2.1% at 10.5 mA cm^−2^ in H‐cell and 92.3 ± 2.4% at 126.7 mA cm^−2^ in flow‐cell, with stability maintained over 150 h. The investigation into CO_2_ electroreduction on BHT‐Cu_0.8_‐Zn_0.2_ catalyst, aided by in situ attenuated total reflection – surface enhanced infrared absorption spectroscopy (ATR‐SEIRAS) and density functional theory (DFT), unveils a reaction mechanism starting with the adsorption of 2CO* intermediate on the catalyst surface, followed by hydrogenation steps leading to the formation of C_2_H_5_OH. Cu and Zn act as bifunctional active sites, enabling optimal binding strength and enhanced catalytic activity toward C_2_H_5_OH production, compared with the single active sites. This work showcases a low overpotential of 0.48 V, which is an ideal performance for industrial production of C_2_H_5_OH using CO_2_RR. We employ the techno‐economic analysis on BHT‐Cu_0.8_‐Zn_0.2_ for CO_2_RR to C_2_H_5_OH and obtain the net present value (NPV) of 8.59 million dollars for the production at industrial scale plant [6] (Tables S1–S5). We further report a carbon efficiency of 73% for converting CO_2_ into C_2_H_5_OH, coupled with an overall energy efficiency of 52% for the full cell. Moreover, the energy cost is estimated at 56.6 GJ per tonne of ethanol, which is the lowest value recorded among current CO_2_ electrolysis systems used for C_2_H_5_OH production (usually 260–1200 GJ/ton C_2_H_5_OH, (Figure 1b; Table S6). Our work highlights the design of bimetallic 2D c‐CP with spatially arranged bi‐active sites, serving as a highly efficient electrocatalyst for sustainable CO_2_ electroreduction, producing value‐added C_2_H_5_OH.

## 2 Results and Discussion

### 2.1 Synthesis and Characterization of Bimetallic 2D c‐CPs

**FIGURE 2:**
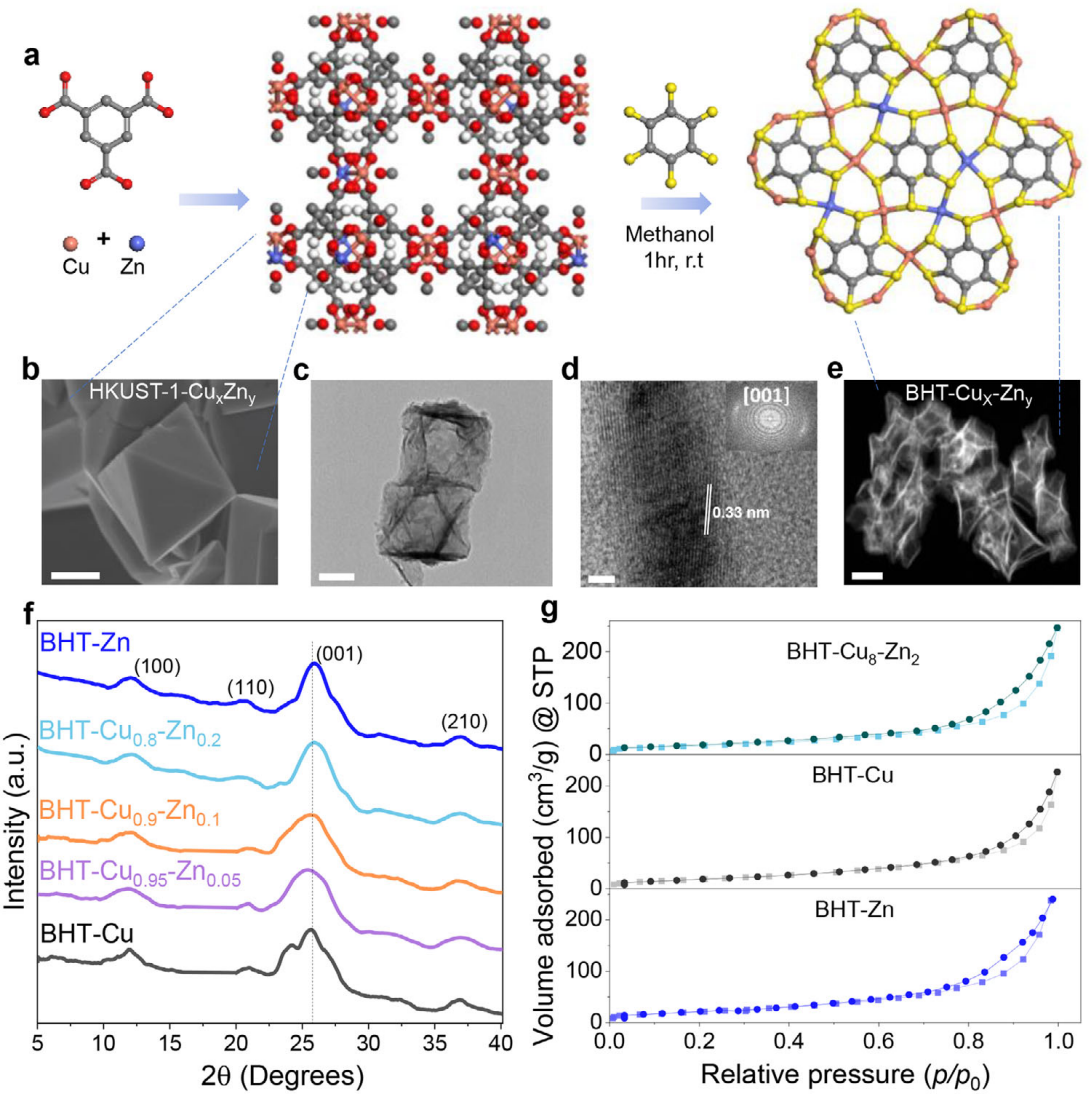
Synthesis and structural characterization of BHT‐Cu_x_‐Zn_y_(x + y = 1) c‐CPs. (a) Synthesis of HKUST‐1‐Cu_x_‐Zn_y_ and their conversion into BHT‐Cu, BHT‐Zn, BHT‐Cu_0.95_‐Zn_0.05_, BHT‐Cu_0.9_‐Zn_0.1_, BHT‐Cu_0.83_‐Zn_0.17_, and BHT‐Cu_0.8_‐Zn_0.2_ c‐CPs. Red: O; grey: C; pink: Cu; blue: Zn; yellow: S. (b) SEM image of HKUST‐Cu_0.8_‐Zn_0.2_, (c–e) TEM, HR‐TEM, and HAADF images of BHT‐Cu_0.8_‐Zn_0.2_, respectively. The inset in Figure 2d is a FFT image. (f) XRD spectra of various BHT‐Cu_x_‐Zn_y_ c‐CPs. (g) Nitrogen adsorption (indicated by a circle symbol with a line) and desorption (indicated by a square symbol with a line) isotherms measured at 77 K for various c‐CPs. Scale bars: 100, 200, 5, and 200 nm for b,c,d, and e, respectively.

The 2D c‐CPs were synthesized through a one‐step process involving MOF to c‐CP transformation, as illustrated in Figure 2a. In a typical STAB procedure, cubic HKUST‐1‐Cu MOF was initially synthesized as previously described [34], and then dispersed in a BHT solution (methanol/water, 7:1 v/v) at room temperature. The HKUST‐1‐Cu c‐CP acted as sacrificial templates and could be completely converted into BHT‐Cu c‐CP after 1 h of reaction. Subsequently, the bimetallic HKUST‐1‐Cu_x_‐Zn_y_ MOFs with varied Cu/Zn ratios were synthesized (Figure 2b), and the STAB strategy was further employed to convert them into BHT‐Cu_x_‐Zn_y_ c‐CPs (synthetic details shown in Methods)_._ Through this strategy, we prepared 5 c‐CPs with spatially separated bimetal centers, including BHT‐Cu_0.95_‐Zn_0.05_, BHT‐Cu_0.9_‐Zn_0.1_, BHT‐Cu_0.83_‐Zn_0.17_, BHT‐Cu_0.8_‐Zn_0.2_, and BHT‐Cu_0.75_‐Zn_0.25_ (Figure 2c, Table S7 and Figure S1–S5). A similar method was also utilized to synthesize BHT‐Zn 2D c‐CP starting from the HKUST‐1‐Zn (Figure S6). The inductively coupled plasma (ICP) measurements presented the metal content of Cu = 36.98 wt.% and Zn = 7.26 wt.% in BHT‐Cu_0.8_‐Zn_0.2_, which were nearly not changed after the 155‐h CO_2_RR test (Cu = 39.20 wt.% and Zn = 8.54 wt.% in the as‐prepared sample). Take the synthetic BHT‐Cu_0.8_‐Zn_0.2_ as an example, the scanning electron microscopy (SEM), the transmission electron microscopy (TEM), and the high‐angle annular dark‐field (HAADF) images present hollow c‐CP with a diameter of c‐CP particles ranging from 400 to 500 nm (Figure 2c–e and Figure S1). The high‐resolution TEM (HR‐TEM) image reveals that the hollow BHT‐Cu_0.8_‐Zn_0.2_ c‐CP are polycrystalline with the lattice fringe showing a *d*‐spacing of 0.33 nm, which can be assigned to the (001) plane (Figure 2d). The XRD measurements indicated that the resultant BHT‐Cu_x_‐Zn_y_ exhibits similar diffraction peaks as BHT‐Cu, with major peaks appearing at 11.8°, 21.5°, 26.3°, and 36.7° assigned to (100), (110), (001), and (210) planes [35, 36], respectively (Figure 2f). The corresponding energy loss spectroscopy (EELS) mapping indicated a homogeneous distribution of Cu, Zn, C, and S elements over the hollow BHT‐Cu_0.8_‐Zn_0.2_ c‐CP (Figure S1 and Table S7). The Brunauer−Emmett−Teller (BET) surface areas of the hollow BHT‐Cu_0.8_‐Zn_0.2_, BHT‐Cu, and BHT‐Zn c‐CP were measured as 79.32, 81.61, and 76.85 m^2^ g^−1^, respectively (Figure 2g). To examine the composition of BHT‐Cu_x_‐Zn_y_ c‐CPs, X‐ray photoelectron spectroscopy (XPS) analysis was conducted, which detected the presence of Cu, Zn, S, and C elements (Figures S7–S9). A high‐resolution XPS scan of a typical Cu 2p 3/2 peak displays a mixed oxidation state of the Cu element with the Cu(I) at 932.6 eV and the Cu (II) at 934.1 eV (Figures S7 and S8). Moreover, the Cu atom near the surface layer or edge of the c‐CP is responsible for the broad peak at 934.1 eV [35]. Furthermore, BHT‐Cu_0.8_‐Zn_0.2_ sample also shows the binding energies for Zn at 1047.5 and 1023.8 for Zn 2p1/2 and 2p3/2, respectively (Figure S9).

### 2.2 Electrochemical CO_2_RR Performance

**FIGURE 3:**
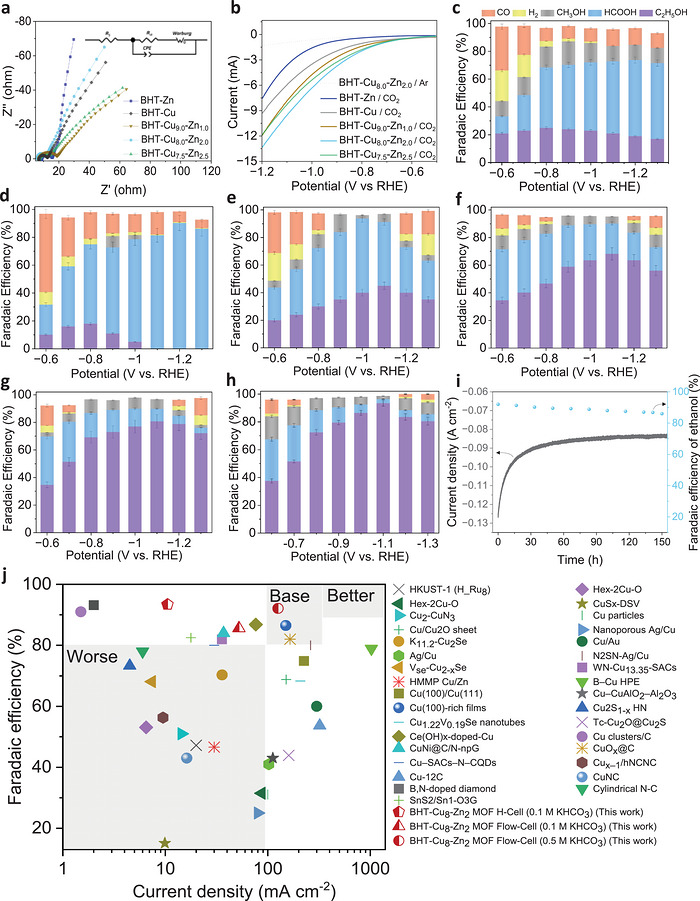
Electrochemical CO_2_RR performance of the BHT‐Cu_x_‐Zn_y_ c‐CPs. (a) EIS spectra of BHT‐Zn, BHT‐Cu, BHT‐Cu_0.9_‐Zn_0.1_, BHT_0.8_‐Cu_0.2_, and BHT‐Cu_0.75_‐Zn_0.25_ (Inset: equivalent electrical circuit.). (b) LSV curves of the c‐CP samples. FE of various gas and liquid products after CO_2_RR with error bars (which represent the standard deviation across multiple independent measurements (*n* ≥ 3)). (c) BHT‐Cu, (d) BHT‐Zn, (e) BHT‐Cu_0.95_‐Zn_0.05_, (f) BHT‐Cu_0.9_‐Zn_0.1_, (g) BHT‐Cu_0.83_‐Zn_0.17_ and (h) BHT‐Cu_0.8_‐Zn_0.2_. (i) Stability tests for BHT‐Cu_0.8_‐Zn_0.2_ in flow‐cell with 0.5 m KHCO_3_ electrolyte. (j) Comparison of the state‐of‐art reported electrocatalysts for C_2+_ alcohols production via electrochemical CO_2_RR.

Electrochemical CO_2_RR performance is a crucial indicator of the effectiveness of an electrocatalyst in transforming CO_2_ into useful chemicals. Essential performance indicators such as FE, overpotential, current density, and product selectivity collectively demonstrate the catalyst's efficacy, energy efficiency, and feasibility for real‐world use in sustainable energy and chemical industrial processes (Figure 3). First, electrochemical impedance spectroscopy (EIS) measurements were conducted over the frequency range of 100 kHz to 100 MHz, and the Nyquist plots were fitted using the equivalent circuit shown in Figure 3a and Figure S18 (The fitted parameters are summarized in Table S12). The circuit includes *R*_s_ (solution resistance), *R*_ct_ (charge transfer resistance), CPE (constant phase element, with *Q* and *n* describing interfacial capacitance), and W (Warburg element, representing diffusion). BHT‐Cu_0.8_–Zn_0.2_ exhibits the lowest *R*_s_ (6.1 Ω) and *R*_ct_ (5.1 Ω), together with reduced diffusion resistance, indicating efficient charge transfer and favorable mass transport compared to the other compositions. These results highlight the superior interfacial properties of BHT‐Cu_0.8_–Zn_0.2_, supporting its enhanced activity in CO_2_ reduction. The electrocatalytic CO_2_RR performances of BHT‐Cu, BHT‐Zn, BHT‐Cu_0.95_‐Zn_0.05_, BHT‐Cu_0.9_‐Zn_0.1_, BHT‐Cu_0.83_‐Zn_0.17_, BHT‐Cu_0.8_‐Zn_0.2_, and BHT‐Cu_0.75_‐Zn_0.25_ were assessed in a gas‐tight H‐cell with CO_2_‐saturated 0.1 m KHCO_3_, and also in flow‐cell with CO_2_‐saturated 0.1 m KHCO_3_ and 0.5 m KHCO_3_ electrolyte, without using any carbon additive (Figure S16). According to linear sweep voltammetry (LSV) measurements, BHT‐Cu, BHT‐Cu_0.9_‐Zn_0.1_, and BHT‐Cu_0.75_‐Zn_0.25_ display comparable current densities of 9.3 to 11.9 mA cm^−2^ (Figure 3b). Meanwhile, BHT‐Cu_0.8_‐Zn_0.2_ shows the highest current density of 13.4 mA cm^−2^ among all the samples, but BHT‐Zn presents a low current density of 7.6 mA cm^−2^ in the CO_2_‐saturated electrolyte.

As is known, the electrochemical CO_2_RR can yield different reduction products such as H_2_, CO, HCOOH, CH_3_OH, and C_2_H_5_OH, which rely on the electron transfer number ranging from 2 to 12. Here, we further utilized ^1^H nuclear magnetic resonance (^1^H NMR), and gas chromatography mass spectrometry (GC‐MS) to detect the reduction products during controlled‐potential electrolysis (CPE) after 155 h of testing (Figures S11–S14). The single‐metallic BHT‐Cu was able to deliver HCOOH (FE = 54.5% at −1.3 V), CO (FE = 33.6% at −0.6 V) and C_2_H_5_OH (FE = 22.5% at −0.8 V (Figure 3c), while the BHT‐Zn majorly generated 2‐electron products such as HCOOH (FE ≈ 90% at −1.2 V), CO and H_2_, along with about 20% FE of C_2_H_5_OH at −0.8 V (Figure 3d). After the incorporation of Zn atoms to replace partial Cu atoms within the CP network, the FE for C_2_H_5_OH production was significantly enhanced while the FE for 2‐electron products such as H_2_ (by suppressing hydrogen evolution reaction), CO, and HCOOH was largely reduced (Figure 3e–h). In particular, the bimetallic BHT‐Cu_0.8_‐Zn_0.2_ displayed the obvious increase of FE of C_2_H_5_OH product in the applied potentials. At an applied potential of −1.1 V, with an overpotential of 0.48 V, the total FE for C_2_H_5_OH) in an H‐cell significantly increased from 53.7 ± 1.6% to 93.4 ± 2.1% as the Cu/Zn ratio was varied from 9.5:0.5 to 8.0:2.0, highlighting the crucial role of Cu/Zn composition in enhancing C_2_H_5_OH selectivity. Notably, the BHT‐Cu_0.8_‐Zn_0.2_ exhibits a FE of 92.3 ± 2.4% for C_2_H_5_OH generation in a flow‐cell at −0.85 V (Figure S17b). But the FE of C_2_H_5_OH was decreased to 73.6% as the Zn ratio was further increased in the BHT‐Cu_0.75_‐Zn_0.25_ sample (Figure S15b). Within the potential range from −0.6 to −1.3 V, the BHT‐Cu_0.8_‐Zn_0.2_ electrocatalyst could also yield limited products of HCOOH, CH_3_OH, and CO. The higher potentials promoted pathways that favor oxygen‐containing intermediates, leading to the formation of C_2_H_5_OH product. Meanwhile, the lower potentials may favor pathways that lead to hydrocarbon formation without oxygen incorporation.

The ^1^H NMR, and GC‐MS‐spectra of the products from BHT‐Cu_0.8_‐Zn_0.2_ after 155 h of testing in an electrolyte saturated with CO_2_ are shown in (Figures S10–S13). Except for DMSO, which was employed as the internal standard, no hydrocarbons could be detected when CO_2_ was substituted with nitrogen (Figure S10), suggesting that the generated alcohols were only transformed from CO_2_. Significant methyl hydrogen peaks of C_2_H_5_OH were observed in the NMR spectra at δ = 1.0 and 3.5 ppm, respectively, after CO_2_ was purged into the electrolyte (Figure S11). Furthermore, to ascertain the origin of the oxygenates produced, CPE measurements were conducted using ^13^CO_2_ as the feedstock. Examination of the liquid products through ^1^H NMR spectroscopy presented three doublets at around 1, 3.6, and 8.2 ppm, indicative of ^13^C‐labeled C_2_H_5_OH and HCOOH, respectively (Figure S12). Gas product analysis using GC‐MS showed signals attributed to ^13^CO (m/z = 29), affirming ^13^C as the exclusive carbon source for all CO_2_RR products (Figure S13). An amount of 9.2 mmol L^−1^ of C_2_H_5_OH was produced based on BHT‐Cu_0.8_‐Zn_0.2_ electrocatalysts after 70 h of CO_2_RR at −1.1 V with the periodic collection of liquid products for C_2_H_5_OH analysis by NMR (Figure S14b). Compared with the other c‐CPs, BHT‐Cu_0.8_‐Zn_0.2_ exhibits a high degree of C_2_H_5_OH selectivity. The electrocatalytic stability of BHT‐Cu, BHT‐Zn, and BHT‐Cu_0.8_‐Zn_0.2_ at −1.1 V was further investigated using the cycloamperometric (i‐t) tests in H‐cell with 0.1 m KHCO_3_ electrolyte and in flow‐cell with 0.5 m KHCO_3_ electrolyte. After a 155‐h CO_2_RR test, the total current density remained almost constant at approximately 9.4 mA cm^−2^ at −1.1 V with C_2_H_5_OH FE of 89% in the H‐cell and 89.7 mA cm^−2^ at −0.85 V with C_2_H_5_OH FE of 86% in the flow‐cell (Figure 3i; Figure S19). Furthermore, the XRD measurement was performed for the hollow BHT‐Cu_0.8_‐Zn_0.2_ c‐CP after 155 h of CO_2_RR at −1.1 V in 0.1 m KHCO_3_, which indicated no obvious changes in the structural integrity (Figure S14c). Consequently, the BHT‐Cu_0.8_‐Zn_0.2_ electrocatalyst exhibited an outstanding FE of 93.4% for C_2_H_5_OH production at room temperature, which is superior to the so‐far reported electrocatalysts (Figure 3j and Table S9).

### 2.3 In Situ ATR‐SEIRAS and Ex Situ XANES

**FIGURE 4:**
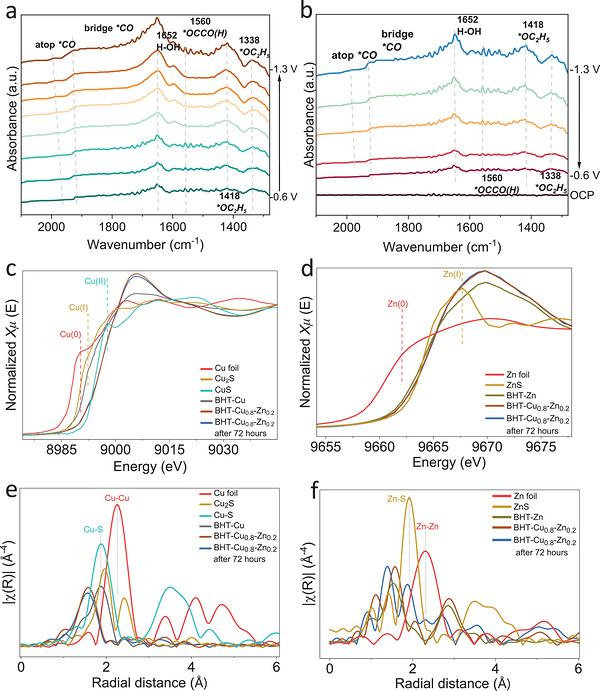
In situ ATR–FTIR and XANES spectra of CO_2_RR. Operando ATR‐SEIRAS spectra were taken by ramping down the applied potential from (a) −0.6 to −1.3 V after 2 h of test, (b) from −1.3 V to −0.6 V after 72 h, and open circuit potential (OCP) in CO_2_‐saturated 0.1 m KHCO_3_. (c) Cu *K*‐edge XANES spectra of BHT‐Cu and BHT‐Cu_0.8_‐Zn_0.2_ samples. (d) Zn *K*‐edge XANES spectra of BHT‐Zn and BHT‐Cu_0.8_‐Zn_0.2_ samples. (e,f) EXAFS spectra of various c‐CPs.

The intermediates during the C_2_H_5_OH generation on BHT‐Cu_0.8_‐Zn_0.2_ were detected by in situ ATR‐SEIRAS (Figure 4a). BHT‐Cu_0.8_‐Zn_0.2_ exhibits two distinct absorption peaks at approximately 2006 and 1934 cm^−1^, corresponding to atop‐adsorbed CO (*CO_atop_) and bridge‐adsorbed CO (*CO_bridge_), respectively. These peaks, observed from −0.6 V (vs RHE) to more negative potentials, display comparable intensities [37, 38]. Prior research suggests that the presence of multiple *CO adsorption configurations enhances C–C coupling on Cu active sites. Consequently, the balanced formation of *CO_atop_ and *CO_bridge_ intermediates on BHT‐Cu_0.8_‐Zn_0.2_ facilitates the production of C_2_ products. The intensity of this peak decreased as the potential increased from −0.6 to −1.3 V and increased when the potential was reduced, indicating the dynamic changes in *CO adsorption behavior. Meanwhile, as the potential increased, all the other peaks associated with the reaction intermediates exhibited enhanced intensity, suggesting that higher potentials promote intermediate formation. Conversely, the reduced intensity was observed with decreasing potential. This observation not only confirms the high stability of the BHT‐Cu_0.8_‐Zn_0.2_ catalyst but also the enhanced catalytic activity toward CO_2_RR, as the intensified peaks indicate a more feasible formation of the reaction intermediates with increased potential (Figure 4a,b). In addition, with increasing potential, a red‐shift of the peak from 2151 to 2140 cm^−1^ was observed, which resulted from the strong electronic interaction between *CO and the coordinated Cu, Zn, and neighboring S atoms [37, 38, 39]. The formation of *CO–CO dimers on BHT‐Cu_0.8_‐Zn_0.2_ was confirmed by the appearance of characteristic peaks at 1560 and 1418 cm^−^^1^, assigned to the *C–O vibration of the *OC–CO(H) intermediate [7]. The enhanced *CO adsorption on BHT‐Cu_0.8_‐Zn_0.2_ suggests that it significantly promotes *CO dimerization, a critical rate‐limiting step in forming C_2_ intermediates [40, 41]. Moreover, the peak at 1560 cm^−1^ was attributed to the stretching of the *OCCOH intermediate, formed via C–C coupling (Figure 4a,b). Additionally, an intensified peak at 1338 cm^−1^ was observed, corresponding to the stretching of surface‐bound *OC_2_H_5_ species [42], a key intermediate in the mechanistic pathway for C_2_H_5_OH formation [42, 43]. This indicates that the C_2_H_5_OH production proceeds through the protonation of *COCOH to form *OCHCOH, followed by subsequent hydrogenation to C_2_H_5_OH.

The Cu coordination environment in bulk BHT–Cu, BHT–Zn, and BHT–Cu_0.8_–Zn_0.2_ was investigated by ex situ X‑ray absorption near edge structure (XANES) and extended X‑ray absorption fine structure (EXAFS). CuS and Cu_2_S were used as references for Cu(II) and Cu(I) [44], respectively. In contrast to the sharp resonance peaks of Cu‑foil and Zn‑foil references, the slightly flattened white line in the Cu K‑edge XANES spectra indicates considerable electron delocalization in BHT–Cu, BHT–Zn, and BHT–Cu_0.8_–Zn_0.2_ (Figure 4c,d). The XANES edge positions confirm that BHT–Cu features Cu(I), and the bimetallic BHT–Cu_x_–Zn_y_ samples retain the same Cu(I) oxidation state even after Zn incorporation. Quantitative EXAFS fitting in R‑space (Figure S21 and Tables S13 and S14) reveals distinct Cu─S coordination shells. BHT–Cu_0.8_–Zn_0.2_ and BHT–Cu display a strong peak at ∼2.1 Å, attributed to tetracoordinated Cu─S bonds, while smaller contributions arise from higher‑shell Cu─C and Cu─Cu scatterings (Figure 4e,f). The fitted coordination numbers (CN ≈ 4) and bond lengths (2.08–2.11 Å) confirm square‑planar Cu–S_4_ motifs, similarly Zn K‑edge spectra also show Zn(II) in square‐planar Zn–S_4_ coordination. Importantly, the major Cu K‑edge peak at 8998 eV remains unchanged after 72 h of CO_2_RR, and the fitted Cu─S coordination persists with only minor adjustments in CN and σ^2^, demonstrating the stability of Cu(I) and the robustness of the Cu–S_4_ environment under electrochemical conditions. Together, the XANES oxidation state analysis and EXAFS fitting provide atomic‑scale evidence for spatially separated Cu–S_4_ and Zn–S_4_ sites within the BHT framework. These distinct local environments rule out alloying or clustering and highlight the cooperative electronic interactions between Cu and Zn centers that underpin the enhanced CO_2_RR performance.

### 2.4 Mechanistic Understanding for CO_2_RR

**FIGURE 5:**
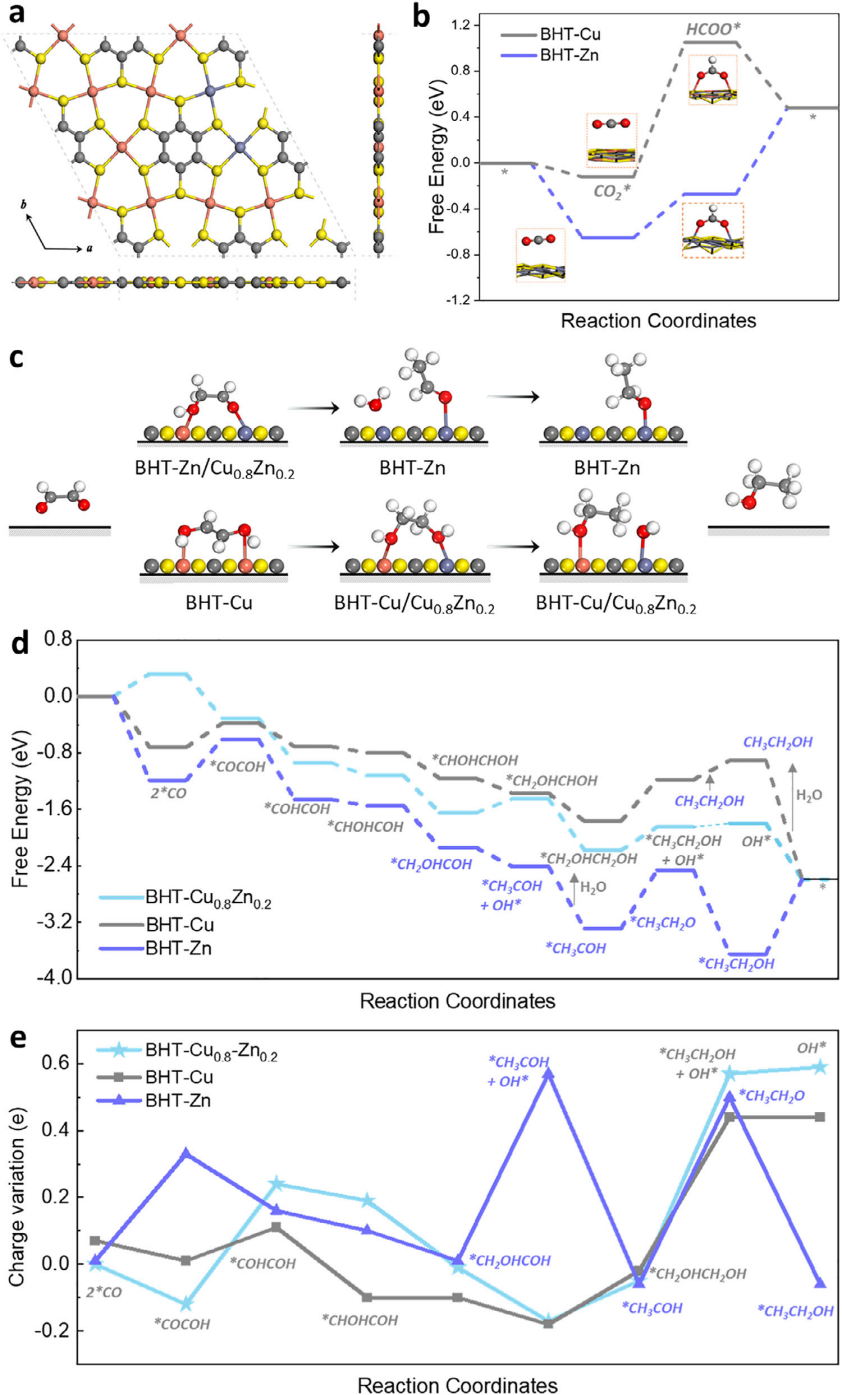
DFT calculation for the understanding of CO_2_RR mechanism. (a) Top and side views of BHT‐Cu_0.8_‐Zn_0.2_, in which the lattice of the 1 × 1 × 1 unit cell is indicated by the grey dashed line. The pink, grey, greyish blue (slate blue), and yellow atoms represent Cu, C, Zn, and S, respectively. (b) Gibbs free energy change profile of CO_2_RR pathway on BHT‐Cu and BHT‐Zn to form HCOOH. (c) Schematic and (d) Gibbs free energy change (ΔG) profile for the CO_2_RR process to form CH_3_CH_2_OH on BHT‐Cu, BHT‐Zn, and BHT‐Cu_0.8_‐Zn_0.2_. (e) Variation in charge transfer from the substrate to the adsorbed species along the CO_2_RR pathway on various c‐CPs.

The above electrochemical CO_2_RR experiments indicated that the CO_2_ could be partially converted to HCOOH based on BHT‐Cu and BHT‐Zn electrocatalysts, but efficiently reduced to yield CH_3_CH_2_OH on BHT‐Cu_0.8_‐Zn_0.2_. Combined with the probing on the intermediate compounds during the CO_2_RR by in situ ATR‐SEIRAS, we further performed the DFT calculations to explore the reaction mechanisms of the CO_2_RR on different catalyst surfaces. The structures of BHT‐Cu, BHT‐Zn, and BHT‐Cu_0.8_‐Zn_0.2_ were first optimized. The optimized BHT‐Cu_0.8_‐Zn_0.2_ structure, with a Cu/Zn ratio of 4:1, features lattice parameters of a = 17.49 Å and b = 17.88 Å (Figure 5a). The Gibbs free energy changes for the CO_2_RR pathway to form HCOOH on the BHT‐Cu and BHT‐Zn catalysts were then calculated (Figure 5b). The CO_2_ was initially physically adsorbed with a large adsorption distance of 2.80 Å away from the catalyst surface, and the adsorption energy values were calculated as −0.12 and −0.65 eV for BHT‐Cu and BHT‐Zn, respectively. The resultant CO_2_* was first reduced to form the intermediate compound HCOO* through the transfer of a proton/electron pair and then converted to HCOOH via a second proton/electron transfer. The Gibbs free energy changes (Δ*G*) for each elementary step were listed in Table S10. As observed, the rate‐determining steps (RDS) on BHT‐Cu and BHT‐Zn correspond to the formation of HCOO*, with Δ*G* values of 1.37 and 0.76 eV, respectively. Additionally, Bader charge analysis was conducted to compare the interactions between the catalyst surfaces and different intermediates, where more transferred electrons indicate stronger binding strength. It is found that the interaction between HCOO* and BHT‐Cu is significantly weaker than that between HCOO* and BHT‐Zn, which reveals the higher catalytic activity of BHT‐Zn toward HCOOH (Figure S20).

The spatially separated Cu and Zn in BHT‐Cu_0.8_‐Zn_0.2_ create Cu/Zn‐S_4_ active sites, providing moderate binding strength with key intermediates, which enhances catalytic activity for C_2_H_5_OH production. Unlike BHT‐Cu and BHT‐Zn, which favor HCOOH formation, BHT‐Cu_0.8_‐Zn_0.2_ facilitates CO dimerization and subsequent hydrogenation steps, leading to improved C_2_H_5_OH selectivity with a lower Δ*G* barrier. Moreover, the C_2_H_5_OH formation was initiated by the intermediate CO*. The CO* could be hydrogenated to yield COH*, which subsequently couples with another CO* to form COCOH* species, with Δ*G* values of 0.34, 0.58, and −0.62 eV for BHT‐Cu, BHT‐Zn, and BHT‐Cu_0.8_‐Zn_0.2_, respectively (Figure 5c,d). The COCOH* carbon sites were further hydrogenated to form *COHCOH species. The distinct interaction strengths of *COHCOH with different catalysts led to different reaction mechanisms for the C_2_H_5_OH production. The BHT‐Zn and BHT‐Cu_0.8_‐Zn_0.2_ exhibited more pronounced charge transfer from metal centers to *COHCOH than BHT‐Cu, which indicated stronger binding of intermediate (Figure 5e). The stronger interaction of intermediate on BHT‐Zn and BHT‐Cu_0.8_‐Zn_0.2_ promoted consecutive reduction of *COHCOH to *CH_2_OHCOH, while the weaker binding on BHT‐Cu favored the formation of *CHOHCHOH (Figure 5c,d). The adsorption strength of *CH_2_OHCOH/*CHOHCHOH on the catalyst surfaces follows the order of BHT‐Zn > BHT‐Cu_0.8_‐Zn_0.2_ > BHT‐Cu. The strong interaction on BHT‐Zn stabilized the C─O bond dissociation of *CH_2_OHCOH to form OH* and *CH_3_COH, which underwent the dynamic adsorption configuration changes from parallel *CH_2_OHCOH to vertical *CH_3_COH (Figure 5d). The *CH_3_COH could be further reduced to *CH_3_CH_2_O, which was stabilized only on BHT‐Zn with a Δ*G* value of 0.83 eV. The *CH_3_CH_2_O could be subsequently reduced to C_2_H_5_OH with another proton/electron transfer. The weaker interactions on BHT‐Cu/Cu_0.8_Zn_0.2_ allowed to facilitate the reduction of *CHOHCHOH/*CH_2_OHCOH to *CH_2_OHCH_2_OH. As the *CH_2_OHCH_2_OH intermediate interacted with another proton/electron pair on BHT‐Cu/Cu_0.8_Zn_0.2_, the *CH_3_CH_2_OH and OH* could be generated and co‐adsorbed on the catalyst surface with C─O bond dissociation. The generated *CH_3_CH_2_OH and OH* were highly stabilized on BHT‐Cu_0.8_‐Zn_0.2_ due to more pronounced interactions (Figure 5c,e), resulting in a less positive Δ*G* of 0.32 eV. For BHT‐Cu/Cu_0.8_Zn_0.2_, the *CH_3_CH_2_OH and OH* finally interacted with two proton/electron pairs to form H_2_O and C_2_H_5_OH. The Δ*G* values for each elementary step across different catalysts are listed in Table S11. In summary, the RDS of CO_2_RR for BHT‐Cu and BHT‐Cu_0.8_Zn_0.2_ is the formation of *CH_3_CH_2_OH and OH* with Δ*G* values of 0.59 and 0.32, respectively. In contrast, for BHT‐Zn, the RDS is the generation of *CH_3_CH_2_O, with a Δ*G* value of 0.83 eV. The interaction strength between the key intermediates (*COHCOH/*CH_2_OHCOH/*CHOHCHOH) and the BHT‐Zn is too strong, but too weak for BHT‐Cu. Therefore, the spatially separated Cu and Zn in BHT‐Cu_0.8_‐Zn_0.2_ act as Cu/Zn‐S_4_ active sites, which endowed moderate binding strength with the adsorbed species and thus led to the superior catalytic activity toward C_2_H_5_OH.

## 3 Conclusions

We reported the successful application of the sacrificial template‐assisted bimetallic synthesis (STAB) strategy to prepare spatially separated Cu‐/Zn‐S_4_ bimetallic 2D c‐CPs. Our work highlighted the promise of BHT‐Cu_0.8_‐Zn_0.2_ 2D c‐CP as an efficient and stable electrocatalyst for the conversion of CO_2_ to C_2_H_5_OH with a record high FE of 92.3 ± 2.4% at 126.7 mA cm^−2^ in a clow‐Cell, with stability maintained over 150 h, paving the way for future developments in sustainable CO_2_ conversion technologies. We utilized in situ ATR‐SEIRAS and other advanced spectroscopic techniques, as well as the theoretical calculation to gain deep insights into the reaction intermediates and pathways during CO_2_RR. The accelerated C_2_H_5_OH production, combined with the suppression of the undesired two‐electron products, such as H_2_ and CO, underscores the potential for Zn and Cu bimetallic systems to optimize product selectivity. Further exploration of the role of Zn atoms in fine‐tuning the catalytic properties could lead to even higher efficiencies and selectivity in CO_2_RR. The BHT‐Cu_0.8_‐Zn_0.2_ electrocatalyst primarily produces C_2_H_5_OH through the hydrogenation and coupling of CO* intermediates, leading to COH, COCOH, and CH2OHCOH species. The spatially separated Cu/Zn active sites in BHT‐Cu_0.8_‐Zn_0.2_ facilitate moderate binding of these intermediates, enhancing catalytic activity toward C_2_H_5_OH formation. In conclusion, the BHT‐Cu_0.8_‐Zn_0.2_ catalyst demonstrates high carbon and energy efficiency meeting industrial standards for CO_2_ to C_2_H_5_OH conversion, with a notably low energy cost compared to other CO_2_ electrolysis systems. Future research should focus on scaling up the synthesis of these bimetallic catalysts while maintaining their performance and stability, as well as optimizing the overall energy efficiency of the process. Continued efforts to reduce energy consumption, alongside the improvements in carbon and energy efficiencies, will be critical in making CO_2_ electroreduction to C_2_H_5_OH a commercially viable and sustainable solution for mitigating carbon emissions.

## Author Contributions

**Renhao Dong** supervised this project and proposed the idea. **Rashid Iqbal** and **Geping Zhang** carried material synthesis and characterizations. **Rashid Iqbal** and **Zhao Yan** carried out electrochemical experiments. **Tianchun Li** and **Yu Jing** carried out DFT calculations. **Rashid Iqbal** conducted in situ ATR‐SEIRAS measurements. **Huan Huang** performed XAS measurements. **Rashid Iqbal**, **Zhao Yan** and **Fengxiang Zhao** carried out scanning electron microscopy and electrochemical impedance spectroscopy measurements. **Rashid Iqbal**, and **Hua Wang** carried out NMR analysis, X‐ray diffraction measurement, X‐ray photoelectron spectroscopy measurement. **Rashid Iqbal**, **Jingcheng Hao**, and **Renhao Dong** co‐wrote the manuscript with the input from all the authors. All authors discussed the results and assisted during manuscript preparation.

## Conflicts of Interest

The authors declare no conflicts of Interest.

## Supporting information




**Supporting File**: smll73914‐sup‐0001‐SuppMat.docx.

## Data Availability

The data that support the findings of this study are available from the corresponding author upon reasonable request.
