# Supplementary material for: Copper‐Zinc Bimetallic Two‐Dimensional Conjugated Coordination Polymers for Highly‐Selective Electrochemical CO2 Reduction to Ethanol
Source: Small. 2026 May 26;22(38):e73914. doi: 10.1002/smll.73914 (PMC13351492; doi:10.1002/smll.73914)
Supplement: Supplementary file 1 — Supporting File: smll73914‐sup‐0001‐SuppMat.docx. [file SMLL-22-e73914-s001.docx]

**Supporting Information**

**Copper-Zinc Bimetallic Two-Dimensional Conjugated Coordination Polymers for Highly-Selective Electrochemical CO_2_ Reduction to Ethanol**

Rashid Iqbal^1,2^, Tianchun Li^3^, Zhao Yan^1,2^, Geping Zhang^1,2^, Fengxiang Zhao^1,2^, Huan Huang^4^, Hua Wang^5^, Yu Jing^3*^, Jingcheng Hao^5*^, Renhao Dong^1,2*^

^1^Department of Chemistry, The University of Hong Kong, Hong Kong 999077, China

^2^Materials Innovation Institute for Life Sciences and Energy (MILES), HKU-SIRI, Shenzhen 518048, China

^3^Jiangsu Co-Innovation Centre of Efficient Processing and Utilization of Forest Resources, College of Chemical Engineering, Nanjing Forestry University, Nanjing 210037, China

^4^Institute of High Energy Physics, Chinese Academy of Sciences, 19B Yuquan Road, Shijingshan District, Beijing 100049, China

^5^Key Laboratory of Colloid and Interface Chemistry of the Ministry of Education, School of Chemistry and Chemical Engineering, Shandong University, Jinan 250100, China

Corresponding authors: [yujing@njfu.edu.cn](mailto:yujing@njfu.edu.cn), [jhao@sdu.edu.cn](mailto:jhao@sdu.edu.cn), and [rhdong@hku.hk](mailto:rhdong@hku.hk)

**Table of Contents**

[**1. Methods** 1](#_Toc227768474)

[1.1 Chemicals 1](#_Toc227768475)

[1.2 Characterisations 1](#_Toc227768476)

[1.3. Experimental Section 2](#_Toc227768477)

[1.4 In-situ ATR-SEIRAS measurements 2](#_Toc227768478)

[1.5 Electrochemical measurements 3](#_Toc227768479)

[4.6 Gas product analysis 4](#_Toc227768480)

[4.7 Liquid product analysis 4](#_Toc227768481)

[4.8 Carbon efficiency (CE) calculation 5](#_Toc227768482)

[4.9 Energy efficiency (EE) calculation 5](#_Toc227768483)

[4.10 *Energy cost (GJ/tonne)* 6](#_Toc227768484)

[4.11 Techno-Economic Analysis (TEA) 6](#_Toc227768485)

[4.12 Computational methods 6](#_Toc227768486)

[4.13 Linear Combination Fitting 7](#_Toc227768487)

[**2. Supplementary Figures and Tables** 8](#_Toc227768488)

[**Supplementary Fig. S1** 8](#_Toc227768489)

[**Supplementary Fig. S2** 9](#_Toc227768490)

[**Supplementary Fig. S3** 10](#_Toc227768491)

[**Supplementary Fig. S4** 11](#_Toc227768492)

[**Supplementary Fig. S5** 12](#_Toc227768493)

[**Supplementary Fig. S6** 13](#_Toc227768494)

[**Supplementary Fig. S7** 14](#_Toc227768495)

[**Supplementary Fig. S8** 15](#_Toc227768496)

[**Supplementary Fig. S9** 16](#_Toc227768497)

[**Supplementary Fig. S10** 17](#_Toc227768498)

[**Supplementary Fig. S11** 18](#_Toc227768499)

[**Supplementary Fig. S12** 19](#_Toc227768500)

[**Supplementary Fig. S13** 20](#_Toc227768501)

[**Supplementary Fig. S14** 21](#_Toc227768502)

[**Supplementary Fig. S15** 22](#_Toc227768503)

[**Supplementary Fig. S16** 23](#_Toc227768504)

[**Supplementary Fig. S17** 24](#_Toc227768505)

[**Supplementary Fig. S18** 25](#_Toc227768506)

[**Supplementary Fig. S19** 26](#_Toc227768507)

[**Supplementary Fig. S20** 27](#_Toc227768508)

[**Supplementary Fig. S21** 28](#_Toc227768508)

[**Supplementary Table 1** 29](#_Toc227768509)

[**Supplementary Table 2** 30](#_Toc227768510)

[**Supplementary Table 3** 31](#_Toc227768511)

[**Supplementary Table 4** 32](#_Toc227768512)

[**Supplementary Table 5** 33](#_Toc227768513)

[**Supplementary Table 6** 34](#_Toc227768514)

[**Supplementary Table 7** 35](#_Toc227768515)

[**Supplementary Table 8** 36](#_Toc227768516)

[**Supplementary Table 9** 37](#_Toc227768517)

[**Supplementary Table 10** 39](#_Toc227768518)

[**Supplementary Table 11** 40](#_Toc227768519)

[**Supplementary Table 12** 41](#_Toc227768520)

[**Supplementary Table 13** 42](#_Toc227768521)

[**Supplementary Table 14** 42](#_Toc227768522)

[**3. References** 43](#_Toc227768523)

# **1.** **Methods**

## 1.1 Chemicals

All chemicals used were at least of analytical grade. Copper nitrate trihydrate (Cu (NO_3_)_2_•3H_2_O), zinc nitrate hexahydrate (Zn (NO_3_)_2_•6H_2_O), zinc acetate, 1,3,5-benzenetricarboxylic acid (H_3_BTC), were purchased from Sigma-Aldrich. Benzene hexathiol (BHT) was purchased from Sigma-Aldrich. Ultrapure water (18.2 MΩ) produced by a Millipore direct-Q system (Millipore) was used throughout the experiments. Commercial reagents were purchased from Sigma-Aldrich (ACS grade) and used as received unless otherwise noted.

## 1.2 Characterisations

Powder X‑ray diffraction data were collected using a Rigaku D/Max 2500 rotating anode X‑ray powder diffractometer using Cu Kα radiation (λ = 1.5406 Å) operated at 1.6 kW (40 kV, 40 mA) power and equipped with a position sensitive detector with at 10.0 mm divergence height slit. Samples were mounted on zero background sample holders by dropping powders from a spatula and then the samples were fixed onto holders with a glass slit. Data were collected in the range of 2*θ* = 2 to 40° with a step of size 0.02° and a scan speed of 1° min^−1^. The in-situ FT-IR spectra were measured using Nicolet iS50, Thermo Fischer scientific, with a universal Zn-Se attenuated total reflection (ATR) accessory in the 500~4000 cm^–1^. Field-emission scanning electron microscope (SEM) was carried out on a (Gemini SEM 500) with an accelerating voltage of 10 kV and a working distance of 5 mm equipped with an energy dispersive spectrometer. Samples were deposited onto a conductive carbon tape and treated by Pt sputtering before observation. High-Resolution Transmission Electron Microscopy (HR-TEM) was performed with (Talos F200X G2）equipped with a field emission gun operated at 200 kV. The sample was suspended in acetone and drop-cast onto a 200-copper mesh, lacey carbon grid (Ted Pella). The concentration of Zn and Cu was determined by optical emission spectrometry (OES) with radial observation of inductively coupled plasma (ICP) using the SPECTRO Arcos spectrometer. The Brunner−Emmet−Teller (BET) method was used to calculate the specific surface area (SBET) using adsorption data at *P*/*P_0_* of 0.05–0.30 with the instrument Jingwei gaobo model number (JWGB-BK200C). The pore size distributions (PSDs) were derived from the adsorption branches of the isotherms using the Barrett-Joyner-Halenda (BJH) model. The total pore volume (V_t_) was estimated from the adsorbed amount at *P*/*P_0_* of 0.995. XPS data were obtained using an ESCALAB 250Xi XPS. Nuclear magnetic resonance (NMR) spectra were recorded on a Bruker AVANCE III HD spectrometer in the given solvents (400 MHz).

## 1.3. Experimental Section

1.3.1 Preparation of the cubic HKUST-1-Cu_x_-Zn_y_

The cubic HKUST-1-Cux-Zny NPs were synthesized as described previously with some modifications^[1]^. Typically, the dissolved H_3_BTC (2.1 g, 0.01 mol) in triethylamine solution (10 mL, 30 wt.% in water) was dried under reduced pressure to obtain BTC triethylammonium salt. Then, the salt was dissolved in distilled water to form a 0.1 M BTC triethylammonium solution. 6.375g NaNO_3_ was dissolved in 1500 mL of a 1:1 (v/v) mixture of ethanol and water to make a 0.05 M NaNO_3_ solution. Then, 15 mL of 0.09 M Cu (NO_3_)_2_•3H_2_O and 0.01 M Zn (NO_3_)_2_•3H_2_O aqueous solution (for HKUST-1-Cu_0.9_-Zn_0.1_ preparation) and 10 mL of 0.1 M BTC triethylammonium solution were added sequentially under vigorously stirring (similarly HKUST-1-Cu, HKUST-1-Zn, HKUST-1-Cu_0.95_-Zn_0.05_, HKUST-1-Cu_0.83_-Zn_0.17_, and HKUST-1-Cu_0.8_-Zn_0.2_). After reaction for an additional 5 minutes at room temperature, the blue HKUST-1 solution was collected by filtration and washed three times with ethanol and water. Finally, the samples were dried at 90 ºC for further use.

1.3.2 Conversion of HKUST-1-Cu_x_-Zn_y_ into hollow BHT-Cu_x_-Zn_y_ c-CPs

Typically, 10 mg as-synthesized HKUST-1-Cu_x_-Zn_y_ was dispersed in 5 mL methanol/water 7:1 (v/v) to form a light blue solution. A solution of 5 mg of BHT in 3 mL of methanol was added to the solution of HKUST-1-Cu_x_-Zn_y_. The reaction solution was placed at room temperature for 1 h under stirring, the dark purple precipitate was filtered and washed with water and acetone. Finally, the samples were vacuum-dried at 90 ºC overnight to remove residual solvent. This method is used to prepare for c-CPs namely, BHT-Cu, BHT-Zn, BHT-Cu_0.95_-Zn_0.5_, BHT-Cu_0.9_-Zn_0.1_, BHT-Cu_0.83_-Zn_0.17_, and BHT-Cu_80.8_-Zn_0.2_ c-CP.

## 1.4 In-situ ATR-SEIRAS measurements

Typically, a thermal evaporator (PuDi vacuum PD-400) was used to evaporate a layer of 100 nm Au film onto the reflecting surface of a Si prism. Before Au deposition, the Si prism was cleaned by sonication in acetone and deionized water baths in succession and polished with 0.05 μm Al_2_O_3_ solution. The above-prepared Au film was coated with catalyst ink using an airbrush to create the functioning electrode. A two-compartment Spectro electrochemical cell with three electrodes. A working electrode, a carbon rod as a counter electrode, and a standard Ag/AgCl electrode serve as the reference electrode in the ATR-SEIRAS measurements. A Fourier Transform Infrared Spectrophotometer (FT-IR, Nicolet iS50, Thermo Fischer Scientific) fitted with a mercury cadmium telluride (MCT) detector was used to obtain every ATR-SEIRAS spectrum. Every electrochemical test was conducted using a CHI electrochemical workstation (CHI760E) under continuous CO_2_ flow conditions in an aqueous solution containing 0.1 M KHCO_3_. Typically, the working electrode was activated initially by performing CV cycles at a scan rate of 0.05 V S^−1^ between -0.1 and -1.4 V versus RHE until the system stabilized. Next, a backdrop of the spectrum at open circuit voltage was gathered. After that, the cathode potential was swept for two minutes at a time, from -0.5 V to -1.3 V versus RHE, to acquire spectra. After subtracting the background, all measurements were made with a spectral resolution of 4 cm^−1^.

## 1.5 Electrochemical measurements

An electrochemical workstation (CHI660E) was used to regulate the CO_2_RR studies in gas-tight H-cell configurations and flow-cell. A Nafion per-fluorinated membrane (Nafion 117，Sinero) served as an ion exchange membrane between the two compartments of the 60 ml H-Cell (Sinero), each holding (anolyte and catholyte) 40 mL of 0.1 M KHCO_3_ where flow-cell ($1\times1$ cm^2^ electrodes area by Sinero) was used with two different concentrations of electrolytes, 0.1 M KHCO_3_ and 0.5 M KHCO_3_ (sigma aldrich). An Ag/AgCl electrode (Sinero) was utilized as the reference electrode and a carbon plate as the counter electrode (Dioxide Materials). Each test was conducted with the reference electrode calibrated. All electrolytes were delivered into the flow electrolytic cell via a peristaltic pump or syringe pump, with the flow rate controlled below 0.1 mL min^-1^. Slurries of catalyst ink were prepared by sonicating 1 milligram of sample powder and 20 μL (5%) Nafion solution (Sinero) in 1 mL of ethanol. In next step, a 1 cm^2^ carbon cloth (Sinero, W0S1011) was drop-casted with 10 μL of the catalyst ink (same for flow-Cell). The working electrode was then completely dried at 50 °C in preparation for further testing. To saturate the electrolyte, CO_2_ was purged into the cathode chamber for at least 60 minutes before the electrochemical test. CO_2_ gas was kept bubbling into the electrolyte during the measurement at a steady flow rate of 0.2 sccm, managed by a digital mass flow controller (SmartTrack100; Sierra). Based on the formula E (*vs.* Reversible Hydrogen Electrode (RHE) = E (*vs.* Ag/AgCl) + 0.059 pH + 0.198, all potentials were converted to RHE. All measurements for carbon efficiency, energy efficiency and energy cost were calculated based on following results (FE = 92 % at 126.7 mA cm^-2^ and CO_2_ flow rate of 0.2 sccm at full-cell potential of 2.08 V). The electrochemical measurements were conducted using a flow-cell or H-cell configuration connected to an electrochemical workstation. The applied potential was systematically varied within the range of –0.6 V to –1.3 V versus the RHE. Potentiostatic electrolysis was performed with incremental negative potential shifts of 0.1 V. At each potential step, electrolysis was maintained for 2 hours, after which NMR analysis was carried out, unless otherwise specified. For long-term stability evaluation, electrolysis was extended up to 150 hours. Throughout the experiments, the steady-state current density was continuously monitored and recorded.

## 4.6 Gas product analysis

Gas products from the cathodic compartment during CO_2_RR were analysed using a GC-2014 (Shimadzu) equipped with a TCD detector and two FID detectors, one of which was coupled with a methanizer to detect low concentration of CO. High purity Ar (99.999%) was used as the carrier gas. The gas products, including H_2_ and CO, were calibrated using standard mixed gases with different concentrations, along with their respective calibration curves. The FEs of the gas products were calculated by the GC data using the following equation:

$$\mathrm{FE}_{G}=\frac{Q_{G}}{Q_{\mathrm{total}}}\times100\%=\frac{\frac{v}{{60s}/\min}\times\frac{y}{{24.5L}/\mathrm{mol}}\times n\times F}{j_{\mathrm{average}}}\times100\%$$

where *v* is the gas flow rate measured by a flowmeter, which is 0.2 sccm for all the tests. *y* is the measured volumetric content of the gas product. *n* is the number of electrons required to form the gas products, and *n* = 2 for H_2_, and CO, respectively. *F* is the Faraday constant (96 485 C mol^−1^). $j_{\mathrm{average}}$ is the average current density.

## 4.7 Liquid product analysis

Liquid products were analysed by a 400 MHz NMR using a pre-saturation technique to suppress the water peak. To perform **^1^H** measurement, 800 *µ*L of electrolyte sampled after CA tests was mixed with 100 *µ*L DMSO standard solution (100 ppm) and 100 *µ*L D_2_O showed the calibration curves of the liquid products, which were plotted by measuring standard solutions containing possible liquid products, including formate, methanol, and ethanol. The faradaic efficiencies of liquid products were calculated as follows:

$$\mathrm{FE}_{L}=\frac{Q_{l}}{Q_{\mathrm{total}}}\times100\%=\frac{n_{L}\times n\times F}{Q_{\mathrm{total}}}\times100\%$$

where $n_{L}$ is the total content of certain liquid products in the catholyte, which was calculated by the concentration and the volume of the catholyte (40 mL).

## 4.8 Carbon efficiency (CE) calculation

*n* is the number of electrons required to form the liquid products, and *n* = 2, 6, and 12 for formate, methanol, and ethanol, respectively. The percentage of CO_2_ converted per total CO_2_ input, which pertains to gas, liquid or a combination of gas and liquid products at 25 °C and 1 ATM, was determined via the following equation:

$$CE of C_{2}H_{5}OH=\frac{\frac{j_{\mathrm{product}} \times60 s}{n \times F}}{\frac{\nu\frac{l}{\min}}{V_{m}}}$$

Where, $j_{\mathrm{product}}$ is the partial current density of ethanol during CO_2_RR (measured in amperes), $\nu$ is the gas flow rate (l min^-1^) and $V_{m}$ is equivalent to 24.05 (l mol^−1^).

## 4.9 Energy efficiency (EE) calculation

The full-cell energy efficiency of ethanol is calculated using the following formula:$EE of C_{2}H_{5}OH=\frac{E_{\mathrm{cell}}^{o}}{E_{full-cell}}\times\mathrm{FE}_{C_{2}H_{5}\mathrm{OH}}\times100$

$$E_{\mathrm{cell}}^{o}=E_{\mathrm{ox}}^{o}- E_{\mathrm{red}}^{o}$$

= 1.23 - 0.09 = 1.14 V

Where, $E_{\mathrm{cell}}^{o}$ represents the thermodynamic cell potential for products ($E_{\mathrm{ethanol}}^{o}$ = 0.09 V, ethanol $E_{\mathrm{cell}}^{o}$ = 1.14 V), ${\Delta G}_{o}$ represents the change in Gibbs free energy for the reaction, *F* is Faraday constant and $E_{full-cell}=2.08 V$ represents the applied cell voltage (non-iR compensated).

## 4.10 *Energy cost (GJ/tonne)*

$$Mass of C_{2}H_{5}OH (tonnes)=\frac{Ethanol Produced (mol) \times Molar Mass of Ethanol (g/mol)}{1,000,000}$$

$$Energy Cost (GJ/tonne)=\frac{Energy Supplied (J)}{Mass of Ethanol (tonnes) \times1,000,000,000}$$

## 4.11 Techno-Economic Analysis (TEA)

A TEA was performed to assess the feasibility of the electrocatalytic process, following the methodology reported in Industrial & Engineering Chemistry Research[2]. The analysis incorporated capital expenditure (reactor design, electrode materials, and balance-of-plant), operating expenditure (electricity consumption, electrolyte usage, catalyst replacement, and maintenance), process efficiency (derived from measured Faradaic efficiencies and current densities), and product yield/selectivity (with ethanol as the primary product). Market assumptions for ethanol pricing and electricity costs were taken from the referenced dataset. The results indicate that catalyst stability, energy efficiency, and product selectivity are the dominant factors influencing process economics, with sensitivity analysis showing that improvements in current density and Faradaic efficiency substantially reduce the cost per unit of ethanol produced. A detailed breakdown of assumptions, input parameters, and cost calculations is provided in the **Supplementary Tables S1-S5** to ensure transparency and reproducibility.

## 4.12 Computational methods

All the spin-polarized computations were performed by using DFT, as implemented in the Vienna ab initio simulation package (VASP)^[3]^. The generalized gradient approximation (GGA) combining with Perdew-Burke-Ernzerhof (PBE) functional was employed to describe the exchange correlation energy. The 2×2×1 supercell of BHT-Cu and BHT-Zn monolayer was utilized for the carbon dioxide reduction reaction (CO_2_RR) simulations. The ion-core electron interactions were treated by the projected augmented wave (PAW) method[4]. The plane wave energy cut-off and Monkhorst-Pack k-space mesh was set to be 400 eV and 3×3×1, respectively. The convergence criteria for atomic relaxation were 5×10^-5^ eV of energy and 0.02 eV Å^-1^ of force, respectively. The van der Waals (vdW) interactions was considered by using DFT-D3 method^[5]^. To avoid the interlayer interactions along the c direction, the vacuum layer was set at 15 Å. All visualization of structures and charge density section plots were carried out by Visualization for Electronic and Structural Analysis (VESTA) software^[6]^. The elementary steps of CO_2_RR involve combined proton and electron transfer, thus the Gibbs free energy (ΔG) of all the elementary steps was calculated based on the computational hydrogen electrode (CHE) model, which defines the free energy of H^+^ + e^-^ as that of 1/2 H_2_ at standard conditions. The ΔG for the elementary steps can be obtained using the following formula:

$$\text{Δ}\text{G}\text{ = Δ}\text{E}\text{ -}{\text{ }\text{T}\text{Δ}\text{S}\text{ + ΔZPE + Δ}\text{G}}_{\text{U}}\text{ -}{\text{ Δ}\text{G}}_{\text{pH}}$$

Where $\text{∆E}$ refers to the difference of total energy obtained from DFT calculations. $\text{∆ZPE}$ and $\text{Δ}\text{S}\text{ }$ refer to the change of zero-point energy and entropy, respectively. The entropies of $\text{H}_{\text{2}}\text{O}$ and $\text{H}_{\text{2}}$ can be obtained directly from the NIST database. T $\text{∆}\text{G}_{\text{U}}\text{ = - }\text{e}\text{U}$ the temperature and is taken as 298.15 K. $\text{∆}\text{G}_{\text{U}}\text{ = - }\text{e}\text{U}$, in which $\text{∆}\text{G}_{\text{pH}}$ is the charge transferred in each elementary step and U is the applied electrode potential. $\text{∆}\text{G}_{\text{pH}}$ represents the Gibbs free energy correction caused by the change of pH and can be $\text{∆}\text{G}_{\text{pH}}\text{ = }\text{k}_{\text{B}}\text{T × ln10 × pH}$ by $\text{k}_{\text{B}}$, where $\text{∆}\text{G}_{\text{pH}}\text{ = }\text{k}_{\text{B}}\text{T × ln10 × pH}$ refers to Boltzmann constant and pH is set to be 0.

## 4.13 Linear Combination Fitting

The linear combination fitting (LCF) analysis was carried out using the ATHENA module within the IFEFFIT software suite, employing reference spectra from Cu foil, CuS, Cu_2_S, ZnS, Zn foil, and the corresponding pristine catalyst powders. This approach enabled quantitative reconstruction of the relative proportions of Cu and Zn species with different oxidation states, and the residual Cu(II). The fitted spectra showed excellent agreement with the experimental XANES profiles, confirming the reliability of the extracted speciation results (**Supporting Figure 21**).

# **2. Supplementary Figures and Tables**

## **Supplementary Fig. S1 |** Energy dispersive spectroscopy (EDS) mapping of BHT-Cu_0.8_-Zn_0.2_ c-CP. (a) STEM-HAADF images, (b) EDS elemental mapping of zinc, (c) copper, (d) carbon, and (e) sulphur.


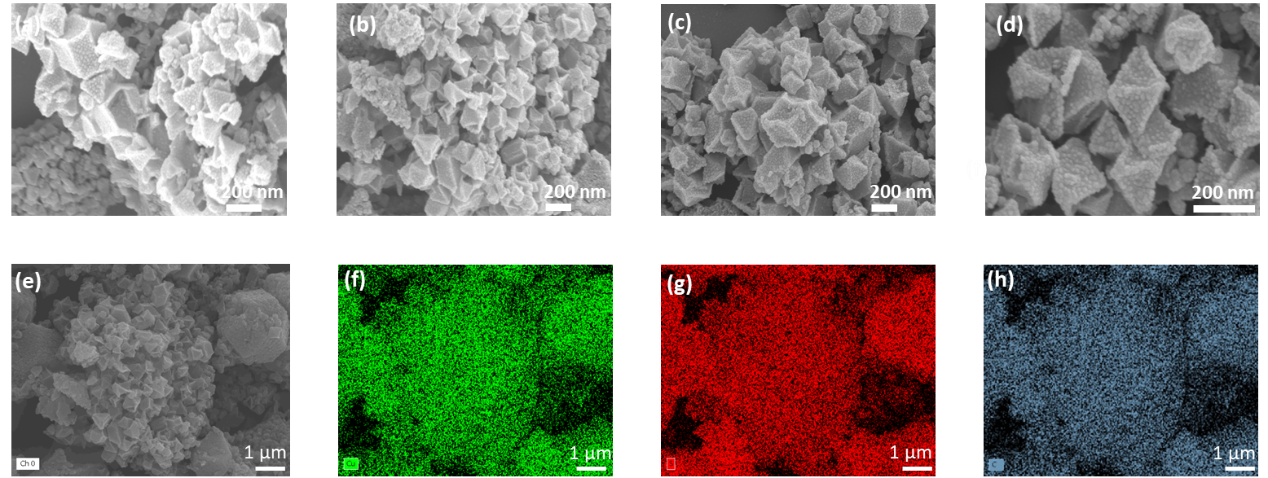


## **Supplementary Fig. S2 |** (a-d) SEM images of hollow BHT-Cu c-CP and (e) mapping images of (f) copper, (g) sulphur and (h) carbon.


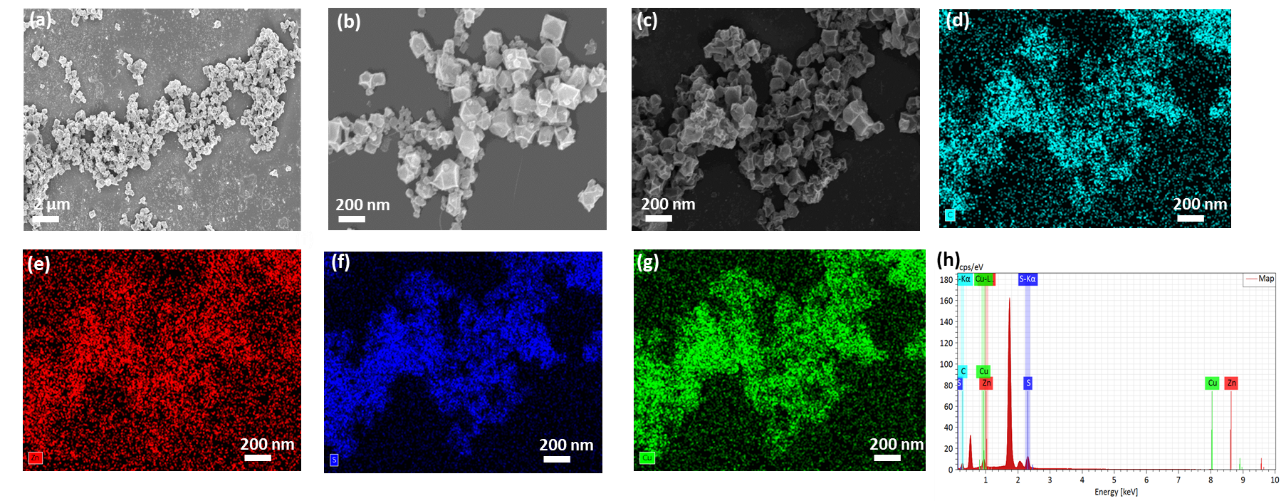


## **Supplementary Fig. S3 |** (a-c) SEM images of hollow BHT-Cu_0.8_-Zn_0.2_ c-CP. SEM mapping images of (d) carbon, (e) zinc, (f) sulphur, (g) copper, and (h) EDX spectra.


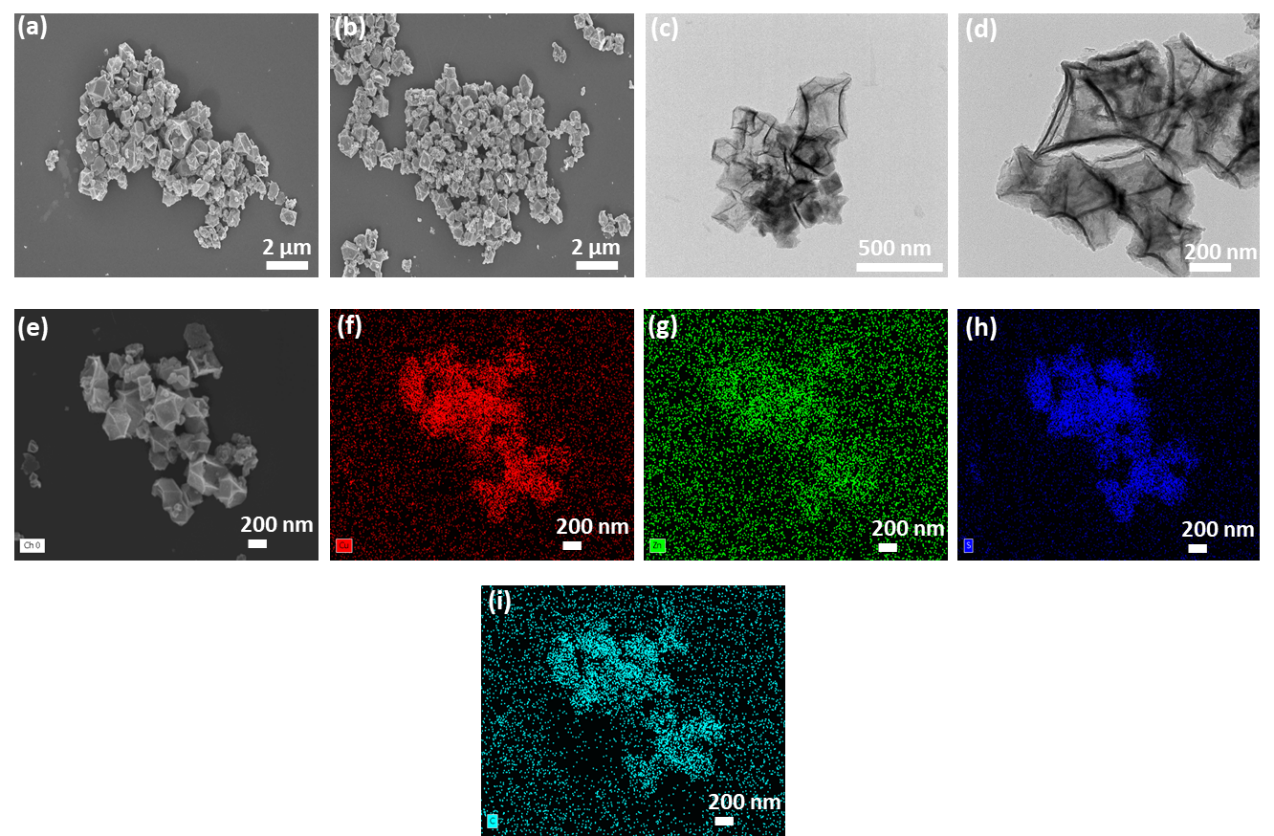


## **Supplementary Fig. S4 |** (a-b) SEM images of hollow BHT-Cu_0.95_-Zn_0.05_ c-CP. (c-d) TEM images. (e) SEM mapping images of (f) copper (g) zinc, (h) sulphur, and (i) carbon.

**
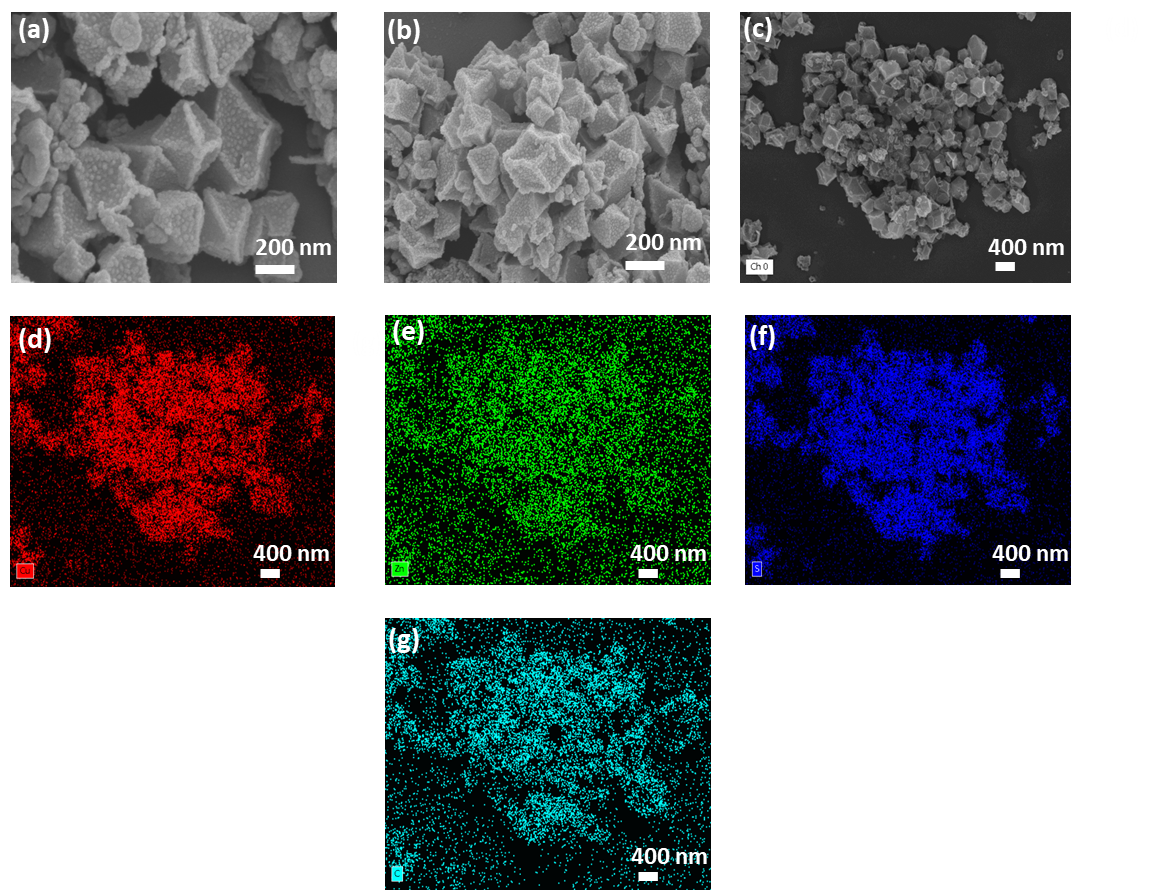
**

## **Supplementary Fig. S5 |** (a-b) SEM images of hollow BHT-Cu_0.9_-Zn_0.1_ c-CP. (c) SEM mapping images of (d) copper (e) zinc, (f) sulphur, and (g) carbon.


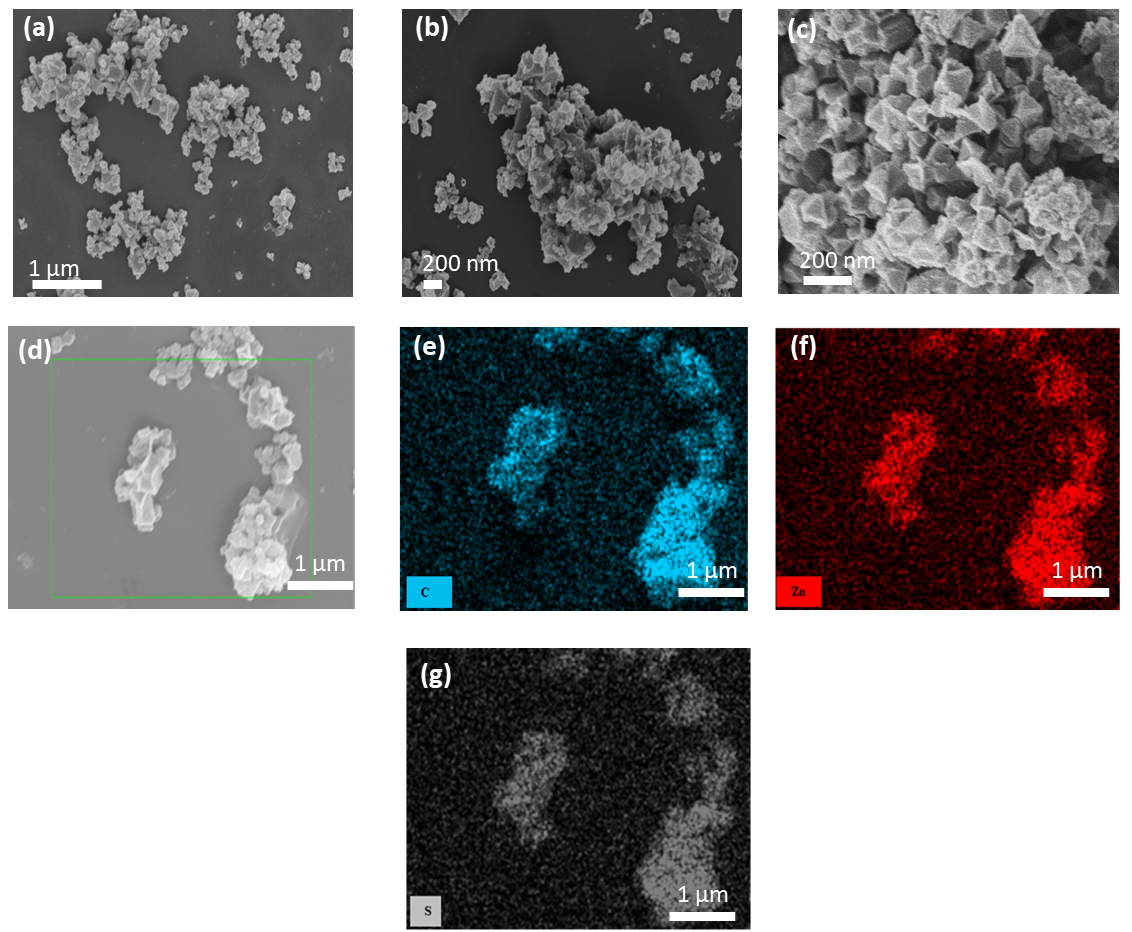


## **Supplementary Fig. S6 |** (a-c) SEM images of hollow BHT-Zn c-CP. (d) SEM mapping images of (e) carbon, (f) zinc, and (g) sulphur.


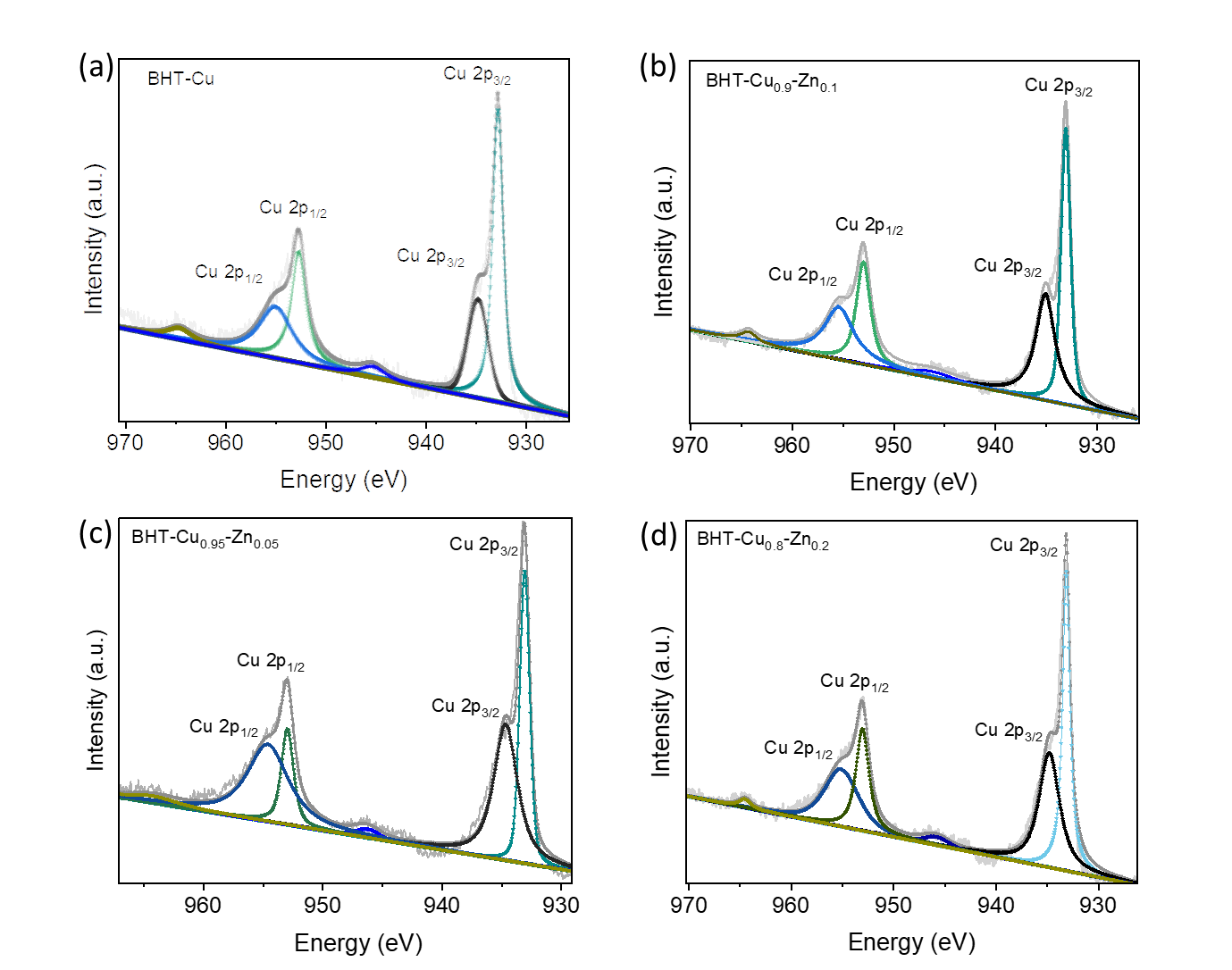


## **Supplementary Fig. S7** **|** XPS deconvoluted spectra of Cu: (a) BHT-Cu with Cu(I)/Cu(II) of 1.25, (b) BHT-Cu_0.9_-Zn_0.1_ with Cu(I)/Cu(II) of 1.15, (c) BHT-Cu_0.95_-Zn_0.05_ with Cu(I)/Cu(II) of 1.23 and (d) BHT-Cu_0.8_-Zn_0.2_ with Cu(I)/Cu(II) of 1.04.


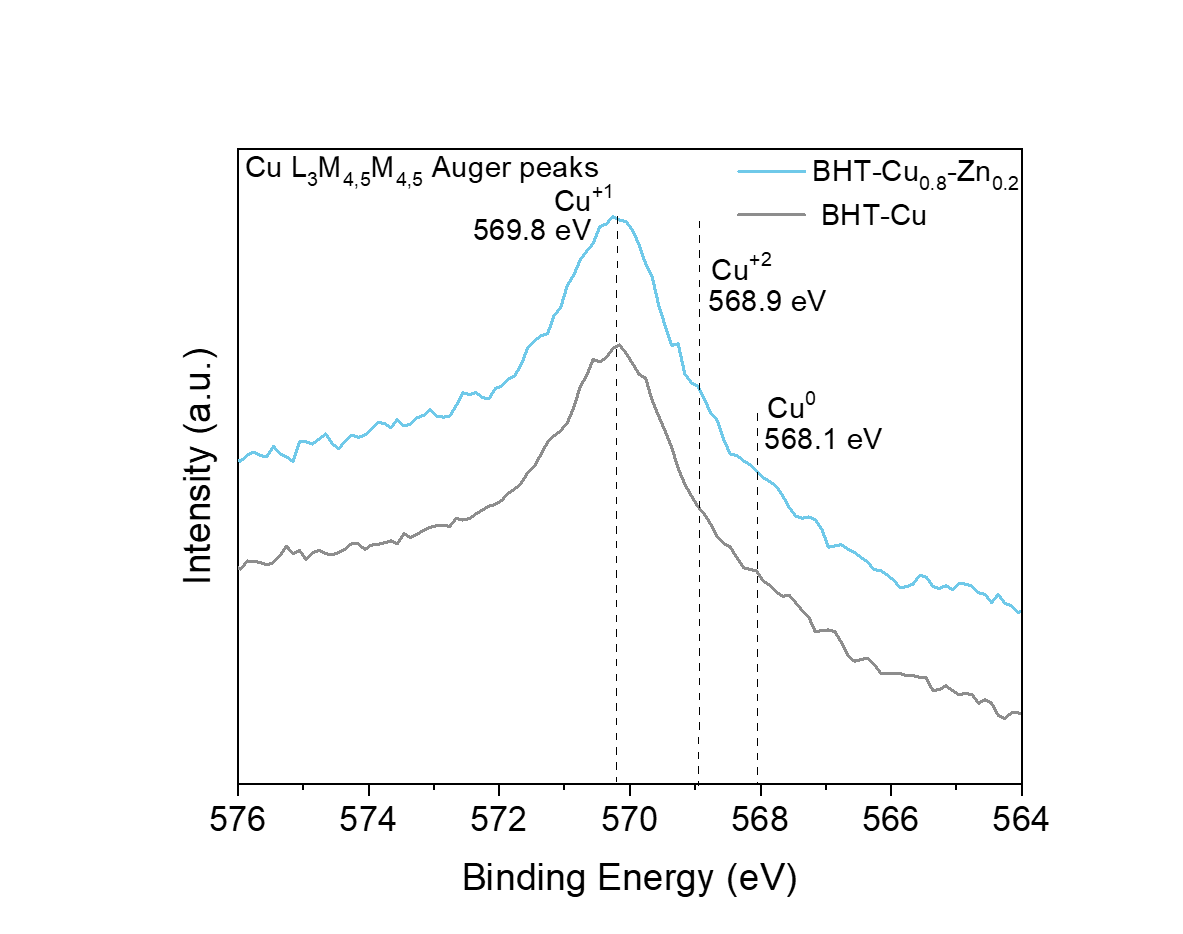


## **Supplementary Fig. S8 |** Cu LMM auger spectrum of BHT-Cu_0.8_-Zn_0.2_ and BHT-Cu.

## **Supplementary Fig. S9** **|** (a) XPS deconvoluted spectra of BHT-Cu_0.8_-Zn_0.2_ for (a) carbon, (b) sulphur, and (c) zinc region.

## **Supplementary Fig. S10 |** ^1^H NMR spectra of hollow BHT-Cu_0.8_-Zn_0.2_ c-CP after nitrogen gas purging in H-type cell at -1.1 V vs RHE in 0.1 M KHCO_3_.

## **Supplementary Fig. S11 |** ^1^H NMR spectra of hollow BHT-Cu_0.8_-Zn_0.2_ c-CP after CO_2_ gas purging at -1.1 V vs RHE in 0.1 M KHCO_3_ after 155 hours in a H-type cell**.**

**
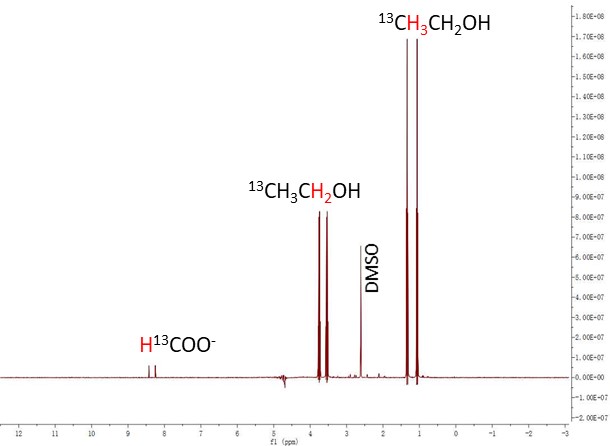
**

## **Supplementary Fig. S12 |** ^1^H NMR spectra of hollow BHT-Cu_0.8_-Zn_0.2_ c-CP after ^13^CO_2_ gas purging at -0.85 V vs RHE in 0.5 M KHCO_3_ after 155 hours in a Flow-Cell**.**

**
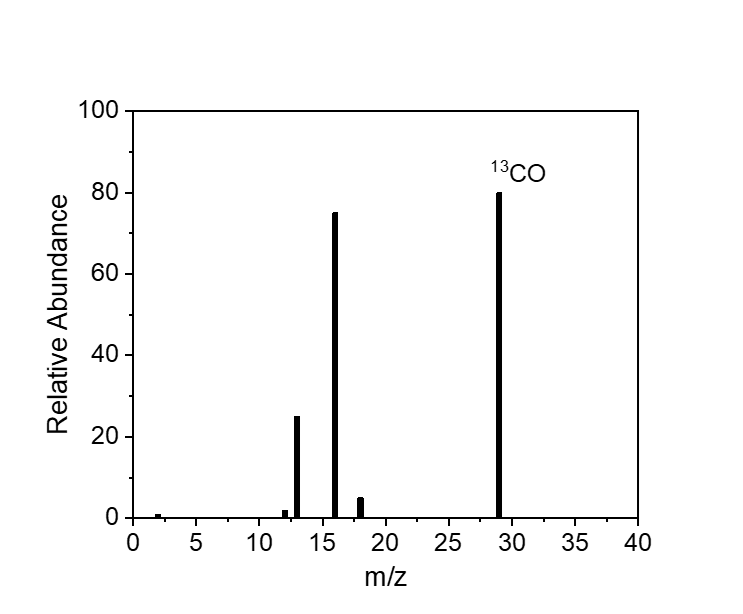
**

## **Supplementary Fig. S13 |** GC-MS-spectra of the gas products produced by isotopic ^13^CO_2_RR -0.85 V vs RHE in 0.5 M KHCO_3_ after 155 hours in a Flow-Cell.

## **Supplementary Fig. S14 |** (a) Calibration curves based on the area of the CH_3_ peak for ethanol NMR spectra with error bars (which represent the standard deviation across multiple independent measurements (n ≥ 3)). (b) ^1^H NMR data points bar graph from the stability test of BHT-Cu_0.8_-Zn_0.2_ c-CP with error bars (which represent the standard deviation across multiple independent measurements (n ≥ 3)). (c) XRD curve of BHT-Cu_0.8_-Zn_0.2_ c-CP after 155 hours of CO_2_RR at -1.1 V vs RHE.


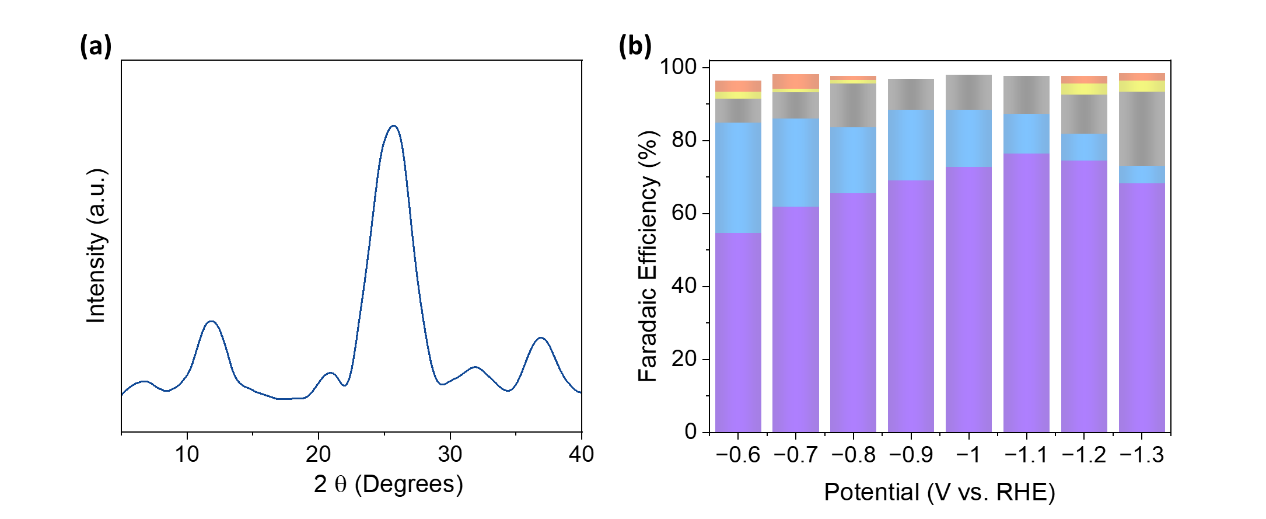


## **Supplementary Fig. S15 |** (a) XRD curve of BHT-Cu_0.75_-Zn_0.25_ before the CO_2_RR tests. (b) FE of BHT-Cu_0.75_-Zn_0.25_ in 0.1 M KHCO_3_ electrolyte.

**
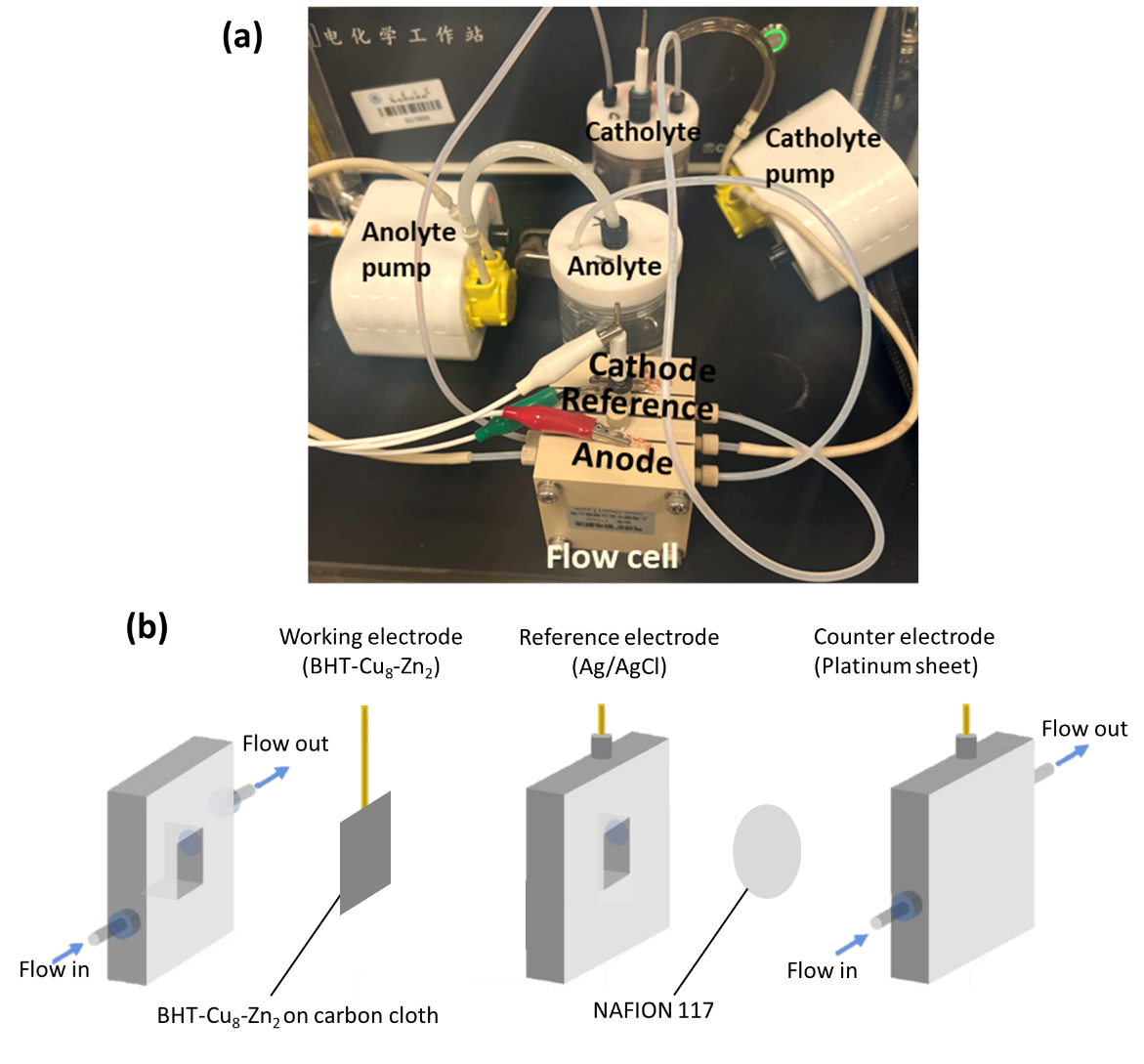
**

## **Supplementary Fig. S16 |** Picture showing the Flow-Cell setup used for CO_2_RR. (b) Scheme of Flow-Cell assembly.


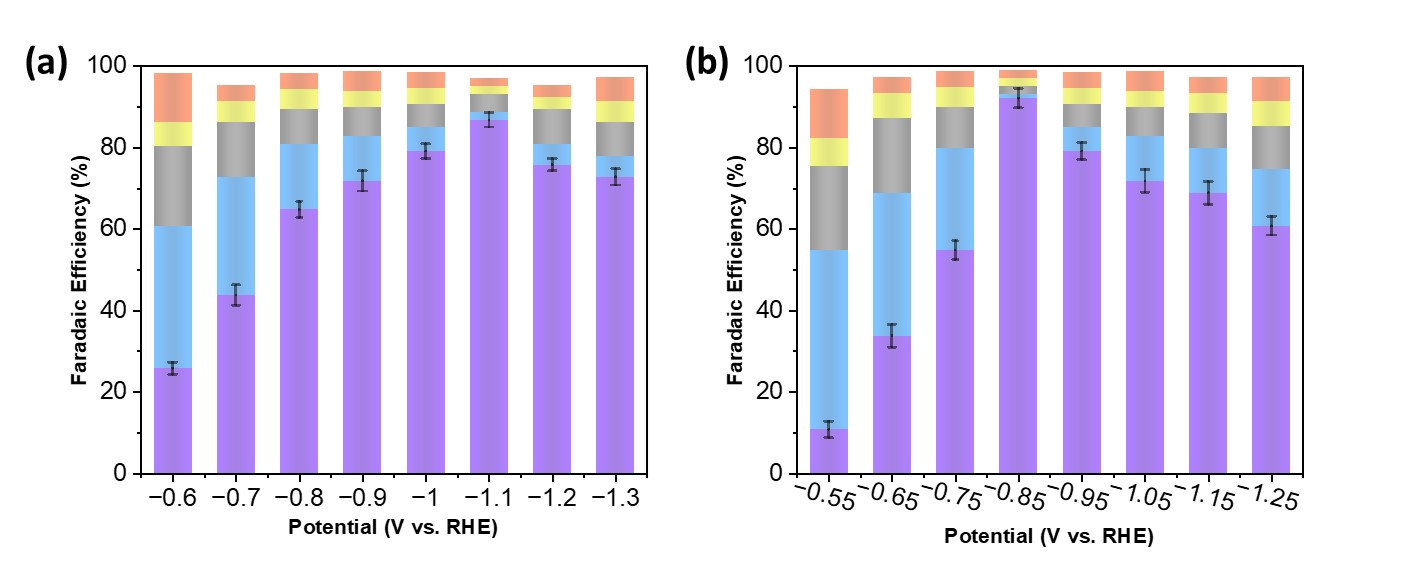


## **Supplementary Fig. S17 |** FE of BHT-Cu_0.8_-Zn_0.2_ c-CP in the Flow-Cell using 0.1 M KHCO_3_ electrolyte. (b) FE of BHT-Cu_0.8_-Zn_0.2_ c-CP in the Flow-Cell using 0.5 M KHCO_3_ electrolyte.

## **Supplementary Fig. S18 | (a)** Equivalent electrical circuit obtained after fitting the electrochemical impedance spectroscopy (EIS) data corresponding to the results shown in Figure b. The circuit consists of Rs (solution resistance), Rct (charge transfer resistance), CPE1 (constant phase element representing double‑layer capacitance and surface heterogeneity), and W1 (finite Warburg element accounting for diffusion processes). The fitted parameters are summarized in Table X, highlighting the influence of Cu/Zn composition on charge transfer and mass transport behaviour. **(b)** Enlarge image of EIS spectra of BHT-Zn, BHT-Cu, BHT-Cu_0.9_-Zn_0.1_, BHT_0.8_-Cu_0.2_, and BHT-Cu_0.75_-Zn_0.25_.


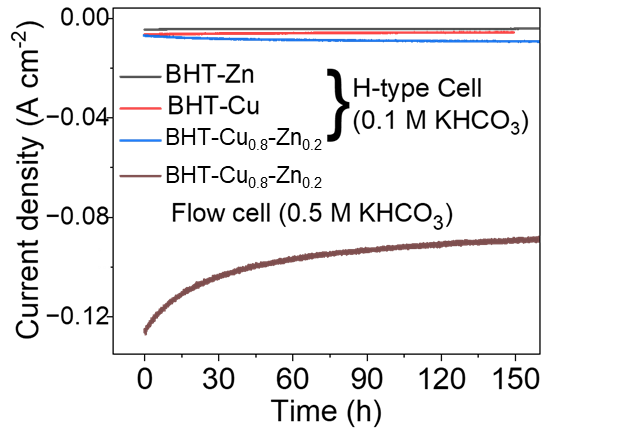


## **Supplementary Fig. S19 |** Stability tests for BHT-Cu_0.8_-Zn_0.2_, BHT-Cu, and BHT-Zn in H-Type-Cell Flow-Cell.


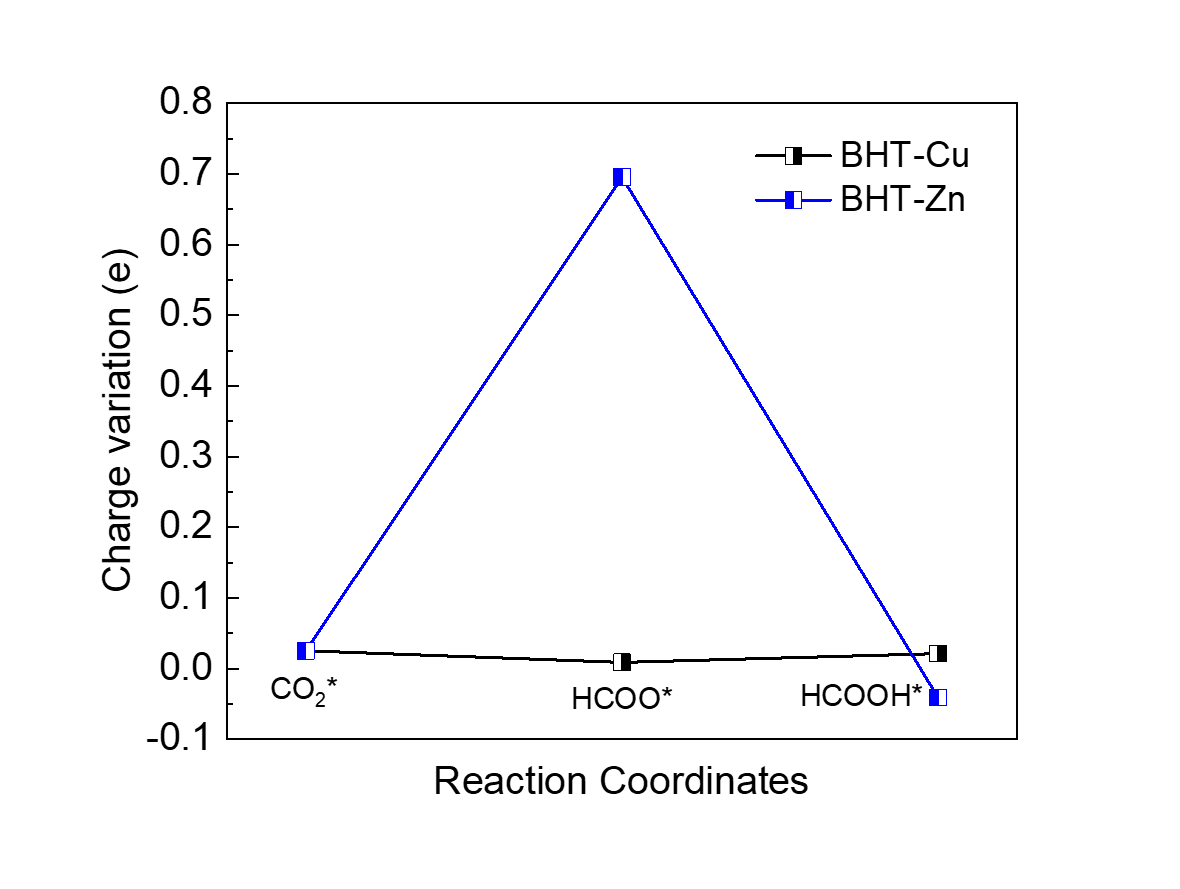


## **Supplementary Fig. S20 |** Variation in charge transfer from the substrate to the adsorbed species along the CO_2_RR pathway to form HCOOH on BHT-Cu and BHT-Zn.

## **Supplementary Fig. S21 | (a)** Cu *K*-edge EXAFS R-space fitting curves for BHT-Cu_0.8_-Zn_0.2_ c-CP before CO_2_RR. (b) Zn *K*-edge EXAFS R-space fitting curves for BHT-Cu_0.8_-Zn_0.2_ c-CP after 72 hours of CO_2_RR, Inset is the fitted model structure of BHT-Cu_0.8_-Zn_0.2_ c-CP.

## **Supplementary Table 1.** Economic Analysis of a CO_2_ Electrolyser System.

| Product production: | 10000 | kg/day |
| --- | --- | --- |
| Product: | Ethanol |  |
| Product Selling Price: | 1.003 | $/kg |
| Product State: | Liquid |  |
| Operating time | 365 | days/year |
| Lifetime | 20 | years |
| Electricity Price | 0.01 | $/kWh |
| **Electrolyser Assumptions:** | | |
| Current Density: | 0.126 | A/cm^2^ |
| Full Cell Voltage: | 2.08 | V |
| Product Selectivity: | 94.86 | % |
| Carbon Conversion: | 73 | % |
| **Electrolyser Balance:** | | |
| # electrons per mole product | 12.0 |  |
| Current Needed: | 3066886.8 | A |
| Electrolyser area: | 2434.0 | m^2^ |
| Power Needed: | 6.6 | MW |
| CO2 needed: | 19105.5 | kg/day |
| CO2 inlet flow rate: | 1090.5 | kg/hr |
| CO2 outlet flow rate: | 294.4 | kg/hr |
|  | 148.7 | m^3^/hr |
| Gas product flow rate: | 0.0 | m^3^/hr |
| Liquid product flow rate: | 0.5 | m^3^/hr |
|  | 8.8 | l/min |
| Electrolyte flow rate: | 88.0 | l/min |
| Hydrogen flow rate: | 0.8 | mol/s |
|  | 70.5 | m^3^/hr |
| Process Water flow: | 3265.3 | gal/day |
| Total gas flow | 219.2 | m^3^/hr |

## **Supplementary Table 2.** Economic Analysis of a CO_2_ Electrolyser System.

| **Capital Costs:** | | |
| --- | --- | --- |
| Electrolyser: | 2238508 | $ |
| Balance of Plant | 1205350 | $ |
| Distillation: | 759485 | $ |
| PSA: | 687476 | $ |
| Total: | 4890820 | $ |
|  |  |  |
| **Operating Costs:** | | |
| Electrolyser Electricity: | 1580 | $/day |
| Maintenance: | 153 | $/day |
| Distillation: | 835 | $/day |
| PSA: | 13 | $/day |
| CO2 Purchase | 764 | $/day |
| Water | 18 | $/day |
| Total: | 3363 | $/day |
|  |  |  |
| Product Income: | 10030 | $/day |
|  |  |  |
| Yearly Profit: | 2433441 | $/yr |
|  |  |  |
| Payback Time: | 2.01 | yr |

## **Supplementary Table 3.** Range of Values for Sensitivity Analysis.

| **Sensitivity Parameters** | **Better** | **Base** | **Worse** |
| --- | --- | --- | --- |
| Electric Price ($/kWh) | 0.02 | 0.03 | 0.04 |
| Selling Price ($/kg) | +15% | Base | -15% |
| Selectivity (%) | 100 | 90 | 80 |
| Voltage (V) | 1.7 | 2 | 2.3 |
| Electrolyser Cost ($/m^2^) | 460 | 920 | 1840 |
| CO_2_ Cost ($/ton) | 0 | 40 | 70 |
| Current Density (mA/cm^2^) | 500 | 300 | 100 |
| Conversion (%) | 70 | 50 | 30 |

## **Supplementary Table 4.** Range of Values for Sensitivity Analysis.

| **Parameter** | **Base Case** | **Optimistic Case** |
| --- | --- | --- |
| Production Rate (ton/day): | 100 | 100 |
| Lifetime (years) | 20 | 20 |
| Operating time (days/year) | 350 | 350 |
| Electricity Price ($/kWh): | 0.05 | 0.03 |
| Current Density (mA/cm^2^): | 200 | 300 |
| Cell Voltage (V): | 2.3 | 2 |
| Product Selectivity (%): | 90 | 90 |
| Conversion (%): | 50 | 50 |
| CO_2_ Price ($/ton): | 70 | 40 |
| Interest Rate (%) | 10 | 10 |
| Electrolyser Cost ($/m^2^) | 1840 | 920 |

## **Supplementary Table 5.** Net Present Value (NPV) is a capital budgeting tool that helps determine if a project's anticipated financial gains.

| Total depreciable capital: | 4,890,820 |  |  |  |  |  |  |  |
| --- | --- | --- | --- | --- | --- | --- | --- | --- |
| Income Tax | 0.300 |  |  |  |  |  |  |  |
| Nominal Interest Rate | 0.100 |  |  |  |  |  |  |  |
|  |  |  |  |  |  |  |  |  |
| Year | Capital Expenses | Working Capital | Depreciation | Net Profit | Net Earning | Discounted Cash Flow | Cash Flow (Present Value) | Cumulative Present Value |
| 0 | -4,890,820 | -244,541 |  |  |  | -5,135,361 | -5,135,361 | -5,135,361 |
| 1 |  |  | -489,082 | 2,433,441 | 2,045,766 | 1,556,684 | 1,415,168 | -3,720,193 |
| 2 |  |  | -880,348 | 2,433,441 | 2,319,652 | 1,439,305 | 1,189,508 | -2,530,685 |
| 3 |  |  | -704,278 | 2,433,441 | 2,196,404 | 1,492,126 | 1,121,056 | -1,409,629 |
| 4 |  |  | -563,422 | 2,433,441 | 2,097,805 | 1,534,382 | 1,048,004 | -361,626 |
| 5 |  |  | -450,934 | 2,433,441 | 2,019,062 | 1,568,129 | 973,685 | 612,059 |
| 6 |  |  | -360,453 | 2,433,441 | 1,955,726 | 1,595,273 | 900,490 | 1,512,549 |
| 7 |  |  | -320,349 | 2,433,441 | 1,927,653 | 1,607,304 | 824,801 | 2,337,350 |
| 8 |  |  | -320,349 | 2,433,441 | 1,927,653 | 1,607,304 | 749,819 | 3,087,169 |
| 9 |  |  | -320,838 | 2,433,441 | 1,927,995 | 1,607,158 | 681,592 | 3,768,761 |
| 10 |  |  | -320,349 | 2,433,441 | 1,927,653 | 1,607,304 | 619,685 | 4,388,447 |
| 11 |  |  | -160,419 | 2,433,441 | 1,815,702 | 1,655,283 | 580,167 | 4,968,613 |
| 12 |  |  |  | 2,433,441 | 1,703,409 | 1,703,409 | 542,759 | 5,511,372 |
| 13 |  |  |  | 2,433,441 | 1,703,409 | 1,703,409 | 493,417 | 6,004,789 |
| 14 |  |  |  | 2,433,441 | 1,703,409 | 1,703,409 | 448,561 | 6,453,349 |
| 15 |  |  |  | 2,433,441 | 1,703,409 | 1,703,409 | 407,783 | 6,861,132 |
| 16 |  |  |  | 2,433,441 | 1,703,409 | 1,703,409 | 370,711 | 7,231,843 |
| 17 |  |  |  | 2,433,441 | 1,703,409 | 1,703,409 | 337,010 | 7,568,854 |
| 18 |  |  |  | 2,433,441 | 1,703,409 | 1,703,409 | 306,373 | 7,875,227 |
| 19 |  |  |  | 2,433,441 | 1,703,409 | 1,703,409 | 278,521 | 8,153,748 |
| 20 |  | 1,222,705 |  | 2,433,441 | 1,703,409 | 2,926,114 | 434,948 | 8588696.062 |
|  |  |  |  |  |  |  | NPV ($millions)= | 8.59 |

## **Supplementary Table 6.** Total energy cost comparison for ethanol production. Comparison of total energy cost for different CO_2_ to ethanol electrolysers. The comparison is limited to electrolysers working at total current densities ≥ 100 mA/cm^2^.

| Full Cell  voltage (V) | EtOH FE (%) | Current  Density  (mA/cm^-2^) | Carbon  Efficiency  (%) | Total energy  cost  (GJ/ton EtOH) | Ref. |
| --- | --- | --- | --- | --- | --- |
| 2.8 | 6 | 100 | 4 | 1004 | [7] |
| 3 | 40 | 900 | 5 | 581 | [8] |
| 3 | 69 | 271 | 5 | 423 | [9] |
| 3.9 | 23 | 300 | 15 | 723 | [10] |
| 3.7 | 41 | 600 | 15 | 412 | [11] |
| 3.5 | 50 | 190 | 7 | 413 | [12] |
| 3.8 | 20 | 200 | 50 | 612 | [13] |
| 3.7 | 21 | 100 | 40 | 586 | [14] |
| 3.5 | 12 | 500 | 70 | 887 | [15] |
| 3.8 | 9 | 100 | 85 | 1187 | [16] |
| 3.7 | 16 | 200 | 75 | 721 | [17] |
| 3.5 | 44 | 200 | 63 | 260.3 | [18] |
| 2.08 | 92.3±2.4 | 126 | 73 | 56.6 | This work |

## **Supplementary Table 7.** Comparison theoretical and practical ratios of the detected metals content by ICP-MS.

| **2D c-CPs** | **Theoretical ratios used for synthesis** | **Practical ratios measured by ICP-MS** |
| --- | --- | --- |
| BHT-Cu_0.95_-Zn_0.05_ | 9.3:0.7 | 9.5:0.5 |
| BHT-Cu_0.9_-Zn_0.1_ | 8.7:1.3 | 9:1 |
| BHT-Cu_0.83_-Zn_0.17_ | 8:2 | 8.3:1.7 |
| BHT-Cu_0.8_-Zn_0.2_ | 7.7:2.3 | 8:2 |
| BHT-Cu_0.75_-Zn_0.25_ | 7.1:2.9 | 7.5:2.5 |

## **Supplementary Table 8.** Table from HRTEM mapping indicating the atomic and weight percentage of hollow BHT-Cu_0.8_-Zn_0.2_ c-CP of various elements.

| Z | Element  Mass | Family | Atomic Fraction (%) | Atomic Error (%) | Mass Fraction (%) | Mass Error (%) | Fit error (%) |
| --- | --- | --- | --- | --- | --- | --- | --- |
| 6 | C | K | 63.12 | 7.38 | 31.21 | 2.56 | 0.64 |
| 16 | S | K | 20.88 | 4.52 | 29.50 | 5.89 | 0.20 |
| 29 | Cu | K | 12.60 | 2.18 | 31.28 | 5.35 | 0.05 |
| 30 | Zn | K | 3.40 | 0.43 | 8.01 | 2.66 | 0.02 |

## **Supplementary Table 9.** Comparison of C_2+_ alcohols production using carbon dioxide reduction.

| **Catalysts** | **Electrolyte** | **Potential (V vs. RHE)** | **Faradaic efficiency (%) and Cell type** | **Current density （mA cm^-2^）** | **References** |
| --- | --- | --- | --- | --- | --- |
| **MOF-based or conjugated polymer materials** | | | | | |
| HKUST-1(H_Ru_8_) | 0.5 M KHCO_3_ | -1.2 V | 47.2% (Ethanol+Methanol) | 20 | [19] |
| **BHT-Cu_0.8_-Zn_0.2_** | **0.1 M KHCO_3_** | **-1.1 V** | **93.4±2.1% (Ethanol in H-Cell)** | **10.5** | **This work** |
| **BHT-Cu_0.8_-Zn_0.2_** | **0.1 M KHCO_3_** | **-1.1 V** | **86.8±1.8% (Ethanol in Flow-Cell)** | **76.4** | **This work** |
| **BHT-Cu_0.8_-Zn_0.2_** | **0.5 M KHCO_3_** | **-0.85 V** | **92.3±2.4% (Ethanol in Flow-Cell)** | **126.7±6.2** | **This work** |
|  | | | | | |
| Hex-2Cu-O | 1 M KOH | -0.66 V | 31.5% (Ethanol+nPrOH in Flow-Cell) | 87 | [20] |
| Hex-2Cu-O | 0.1 M KHCO_3_ | -1.2 V | 53.1% (Ethanol+nPrOH in H-Cell) | 6.5 | [20] |
| **Cu metal and hybrid materials** | | | | | |
| CuSx-DSV | 0.1 M KHCO_3_ | –0.92 V | ~15.1% (PrOH) (Flow-Cell) | 9.9 | [21] |
| Cu_2_-CuN_3_ | 0.1 M KHCO_3_ | -1.2 V | 51% (Ethanol) (H-Cell) | 14.4 | [22] |
| Cu particles | 2 M KCl | -1.2 V | 31.1%(Ethanol) (Flow-Cell) | ~100 | [23] |
| Cu/Cu_2_O sheet | 2 M KOH | - 0.8 V | 68.8% (Ethanol) (Flow-Cell) | 151 | [24] |
| Nanoporous Ag/Cu | 1 M KOH | -0.7 V | 25% (Ethanol)  (Flow-Cell) | 80 | [25] |
| K_11.2_-Cu_2_Se | 0.1 M KHCO_3_ | −0.4 V | 70.3(Ethanol) (H-Cell) | 35.8 | [26] |
| Cu/Au | 1 M KOH | -0.75 V | 60% (Ethanol)  (Flow-Cell) | 300 | [27] |
| Binding-site diverse Ag/Cu | 1 M KOH | –0.67 V | ~41% (Ethanol)  (Flow-Cell) | 102.5 | [28] |
| N_2_SN-functionalized Ag–Cu | 0.1 M KHCO_3_ | -4.5V (MEA electrolysers) | > 80% (Ethanol)  (Flow-Cell) | 261 | [29] |
| V_Se_-Cu_2-x_Se | 0.5 M KHCO_3_ | -0.8 V | 68.1% (Ethanol)  (Flow-Cell) | 7.4 | [30] |
| WN-Cu_13.35_-600-SACs | 0.1 CsHCO_3_ | -1.1 V | 82% (Ethanol)  (Flow-Cell) | 35.6 | [31] |
| HMMP Cu/Zn alloys | 0.1 M KHCO_3_ | -0.80 V | 46.6% (Ethanol)  (H-Cell) | 30 | [32] |
| B–Cu HPE | 0.1 M KHCO_3_ | -0.9 V | 78.9% (52.4% Ethanol, 20% Ethylene, 6.7% n-propanol)  (Flow-Cell) | 1025 | [33] |
| Cu (100)/Cu (111)  interface | 1 M KHCO3 | -0.9 V | 74.9 ± 1.7%  (Flow-Cell) | 224.7 | [34] |
| Cu–CuAlO_2_–Al_2_O_3_  (Cu/Al = 3:1) | 0.1 M KHCO_3_ | -1.2 V | 85.6% (71.1% of Ethylene and 14.5% and Ethanol)  (Flow-Cell) | 52.3 | [35] |
| Cu (100)-rich films | 2 M KOH | -0.95 V | 86.5 (Ethanol)  (Flow-Cell) | 150 | [36] |
| Cu_2_S_1-x_ HN | 0.5 M KHCO_3_ | -0.3 V | 73.3% (Ethanol)  (Flow-Cell) | 4.5 | [37] |
| Cu_1.22_V_0.19_Se nanotubes | 0.1 M KHCO_3_ | -0.80 V | 68.3% (Ethanol)  (Flow-Cell) | 207.9 | [38] |
| Tc-Cu_2_O@Cu_2_S_12h_ | 0.5 M KHCO_3_ | -0.65 V | 43.9% (Ethanol)  (Flow-Cell) | 160 | [39] |
| Ce (OH)x-doped-Cu | 1 M KOH | -0.7 V | 43% (Ethanol)  (H-Cell) | 112 | [40] |
| **Cu-based Carbon materials** | | | | | |
| Cu clusters on ­Oxidized Carbon | 0.1 M KHCO_3_ | –0.7 V | 91% (Ethanol)  (H-Cell) | 1.5 | [41] |
| CuNi@C/N-npG | 0.5 M KHCO_3_ | −0.78 V | 84% (Ethanol)  (Flow-Cell) | 37 | [42] |
| CuOx@C | 0.1 M KHCO_3_ | -1.0 V | 82 % (47% Ethanol, 35% Ethylene)  (Flow-Cell) | 165 | [43] |
| Cu–SACs–N–CQDs | 0.1 M KHCO_3_ | -0.2 V | > 80% (Ethanol)  (Flow-Cell) | 30 | [44] |
| Cu_–1_/hNCNC | 1 M KOH | -0.3 V | 56.3% (Ethanol)  (Flow-Cell) | 9.5 | [45] |
| Cu-12C | 1 M KOH | -1.2 V | 53.7% (Ethanol)  (Flow-Cell) | 321 | [46] |
| CuNC | 0.1 M CsHCO_3_ | −0.5 V | 43% (Ethanol)  (Flow-Cell) | 16.2 | [47] |
| **Carbon material** | | | | | |
| B,N-doped  nano diamond | 0.1 M NaHCO_3_ | -1.1 V | 93.2 (Ethanol)  (H-Cell) | 0.8 | [48] |
| Cylindrical mesoporous N-doped Carbon | 0.1 M KHCO_3_ | −0.4 V | 78% (Ethanol)  (H-Cell) | 6.0 | [49] |
| **Other metallic material** | | | | | |
| SnS_2_/Sn_1_-O3G | 0.5 M KHCO_3_ | -0.9 V | 82.5% (Ethanol)  (Flow-Cell) | 17.8 | [50] |

## **Supplementary Table 10.** The DG (eV) of each elementary step to form HCOOH on BHT-Cu and BHT-Zn.

| The elementary step | BHT-Cu (eV) | BHT-Zn (eV) |
| --- | --- | --- |
| CO_2_* + H^+^ + e^-^ → HCOO* | 1.37 | 0.40 |
| HCOO* + H^+^ + e^-^ → HCOOH +* | -0.74 | 0.76 |

## **Supplementary Table 11.** The DG (eV) of each elementary step to form CH_3_CH_2_OH on BHT-Cu, BHT-Zn and BHT-Cu_0.8_-Zn_0.2_.

| The elementary step | BHT-Cu (eV) | BHT-Zn (eV) | BHT-Cu_8_Zn_2_ (eV) |
| --- | --- | --- | --- |
| 2CO* + H^+^ + e^-^ → COCOH* | 0.34 | 0.58 | -0.62 |
| COCOH* + H^+^ + e^-^ → COHCOH* | -0.33 | -0.85 | -0.62 |
| COHCOH* + H^+^ + e^-^ → CHOHCOH* | -0.09 | -0.08 | -0.18 |
| BHT-Cu: CHOHCOH* + H^+^ + e^-^ → CHOHCHOH*/  BHT-Zn/Cu_0.8_Zn_0.2_: CHOHCOH* + H^+^ + e^-^ → CH_2_OHCOH | -0.36 | -0.59 | -0.53 |
| BHT-Cu/Cu_0.8_Zn_0.2_: CHOHCHOH*/CH_2_OHCOH* + H^+^ + e^-^ → CH_2_OHCHOH*  BHT-Zn: CH_2_OHCOH* + H^+^ + e^-^ → CH_3_COH* + OH* | -0.21 | -0.27 | 0.20 |
| BHT-Cu/Cu_0.8_Zn_0.2_: CH_2_OHCHOH* + H^+^ + e^-^ → CH_2_OHCH_2_OH*  BHT-Zn: OH* + H^+^ + e^-^ → H_2_O + * | -0.39 | -0.88 | -0.73 |
| BHT-Cu/Cu_0.8_Zn_0.2_: CH_2_OHCH_2_OH* + H^+^ + e^-^ → CH_3_CH_2_OH* + OH*  BHT-Zn: CH_3_COH* + H^+^ + e^-^ → CH_3_CH_2_O* | 0.59 | 0.83 | 0.32 |
| BHT-Cu/Cu_0.8_Zn_0.2_: OH* + H^+^ + e^-^ → H_2_O + *  BHT-Zn: CH_3_CH_2_O* + H^+^ + e^-^ → CH_3_CH_2_OH + * | -1.68 | -0.13 | -0.79 |

## **Supplementary Table 12.** Electrochemical impedance spectroscopy (EIS) fitting parameters for BHT–Cu-Zn c-CPs samples with varying Cu/Zn ratios. R_s_ represents the solution resistance, R_ct_ the charge transfer resistance, CPE1‑Q and CPE1‑n describe the constant phase element (pseudo‑capacitance and exponent), and W1 parameters (R, T, P) correspond to diffusion resistance, diffusion time constant, and phase factor of the Warburg element. The values highlight the influence of Cu/Zn composition on interfacial charge transfer and mass transport behaviour.

| **Sample** | **Rs (Ω)** | **Rct (Ω)** | **CPE1‑Q F⋅s^(𝑛−1)^** | **CPE1‑n (dimensionless)** | **Warburg W1‑R (Ω)** | **Warburg**  **W1‑T (s)** | **Warburg**  **W1‑P (dimensionless)** |
| --- | --- | --- | --- | --- | --- | --- | --- |
| **BHT-Cu_0.8_-Zn_0.2_** | 6.10 | 5.12 | 3.20 × 10⁻⁵ | 0.878 | 187.7 | 7.587 s | 0.662 |
| **BHT-Cu_0.9_-Zn_0.1_** | 6.52 | 11.81 | 2.81 × 10⁻⁵ | 0.90 | 78.6 | 2.54 | 0.616 |
| **BHT-Cu_0.75_-Zn_0.25_** | 7.63 | 5.98 | 3.54 × 10⁻⁵ | 0.87 | 119.5 | 5.2 | 0.580 |
| **BHT-Zn** | 6.23 | 10.90 | 5.48 × 10⁻⁵ | 0.787 | 159.2 | 9.634 s | 0.538 |
| **BHT-Cu** | 9.122 | 5.80 | 2.44 × 10⁻⁵ | 0.931 | 3.646 | 0.041 s | 0.431 |

## **Supplementary Table 13.** The EXAFS fitting results of R -space for BHT–Cu_0.8_-Zn_0.2_ c-CPs before CO_2_RR.

| **Path** | **Coordination Number (CN)** | **Bond length R (Å)** | **σ² (10⁻³ Å²)** | **R‑factor** |
| --- | --- | --- | --- | --- |
| **Cu–S1** | 4.81 ± 0.15 | 2.08 ± 0.01 | 38.9 ± 3.5 | 0.0119 |
| **Cu–S2** | 4.33 ± 0.21 | 1.22 ± 0.03 | 348 ± 31 | 0.0082 |
| **Cu–S3** | 4.43 ± 0.04 | 2.59 ± 0.02 | 53.7 ± 1.5 | 0.0085 |

## **Supplementary Table 14.** The EXAFS fitting results of R -space for BHT–Cu_0.8_-Zn_0.2_ c-CPs after CO_2_RR of 72 hours.

| **Path** | **Coordination Number (CN)** | **Bond length R (Å)** | **σ² (10⁻³ Å²)** | **R‑factor** |
| --- | --- | --- | --- | --- |
| **Cu–S1** | 3.47 ± 0.03 | 2.11 ± 0.02 | 13.2 | 0.0016 |
| **Cu–S2** | 3.95 ± 0.14 | 3.36 ± 0.02 | 48.6 | 0.0032 |
| **Cu–S3** | 4.1 ± 0.16 | 2.39 ± 0.02 | 35.4 | 0.0060 |

# **3. References**

[1] C. Huang, W. Sun, Y. Jin, Q. Guo, D. Mücke, X. Chu, Z. Liao, N. Chandrasekhar, X. Huang, Y. Lu, G. Chen, M. Wang, J. Liu, G. Zhang, M. Yu, H. Qi, U. Kaiser, G. Xu, X. Feng, R. Dong, A General Synthesis of Nanostructured Conductive Metal–Organic Frameworks from Insulating MOF Precursors for Supercapacitors and Chemiresistive Sensors, Angewandte Chemie International Edition, n/a e202313591.

[2] M. Jouny, W. Luc, F. Jiao, General Techno-Economic Analysis of CO_2_ Electrolysis Systems, Industrial & Engineering Chemistry Research, 57 (2018) 2165-2177.

[3] J.P. Perdew, K. Burke, M. Ernzerhof, Generalized gradient approximation made simple, Physical review letters, 77 (1996) 3865.

[4] G. Kresse, D. Joubert, From ultrasoft pseudopotentials to the projector augmented-wave method, Physical review b, 59 (1999) 1758.

[5] L. Wang, T. Maxisch, G. Ceder, Oxidation energies of transition metal oxides within the GGA+ U framework, Physical Review B, 73 (2006) 195107.

[6] J. Rossmeisl, A. Logadottir, J.K. Nørskov, Electrolysis of water on (oxidized) metal surfaces, Chemical physics, 319 (2005) 178-184.

[7] F. Li, Y.C. Li, Z. Wang, J. Li, D.-H. Nam, Y. Lum, M. Luo, X. Wang, A. Ozden, S.-F. Hung, Cooperative CO2-to-ethanol conversion via enriched intermediates at molecule–metal catalyst interfaces, Nature Catalysis, 3 (2020) 75-82.

[8] M.A. Adnan, A. Shayesteh Zeraati, S.K. Nabil, T.A. Al-Attas, K. Kannimuthu, C.-T. Dinh, I.D. Gates, M.G. Kibria, Directly-Deposited Ultrathin Solid Polymer Electrolyte for Enhanced CO2 Electrolysis, Advanced Energy Materials, 13 (2023) 2203158.

[9] L. Shang, X. Lv, L. Zhong, S. Li, G. Zheng, Efficient CO_2_ electroreduction to ethanol by Cu3Sn catalyst, Small Methods, 6 (2022) 2101334.

[10] X. Wang, Z. Jiang, P. Wang, Z. Chen, T. Sheng, Z. Wu, Y. Xiong, Ag+‐Doped InSe Nanosheets for Membrane Electrode Assembly Electrolyzer toward Large‐Current Electroreduction of CO_2_ to Ethanol, Angewandte Chemie International Edition, 62 (2023) e202313646.

[11] C.M. Gabardo, C.P. O’Brien, J.P. Edwards, C. McCallum, Y. Xu, C.-T. Dinh, J. Li, E.H. Sargent, D. Sinton, Continuous carbon dioxide electroreduction to concentrated multi-carbon products using a membrane electrode assembly, Joule, 3 (2019) 2777-2791.

[12] Z. Gu, H. Shen, Z. Chen, Y. Yang, C. Yang, Y. Ji, Y. Wang, C. Zhu, J. Liu, J. Li, Efficient electrocatalytic CO_2_ reduction to C_2+_ alcohols at defect-site-rich Cu surface, Joule, 5 (2021) 429-440.

[13] R.K. Miao, Y. Xu, A. Ozden, A. Robb, C.P. O’Brien, C.M. Gabardo, G. Lee, J.P. Edwards, J.E. Huang, M. Fan, Electroosmotic flow steers neutral products and enables concentrated ethanol electroproduction from CO_2_, Joule, 5 (2021) 2742-2753.

[14] Y. Xu, R.K. Miao, J.P. Edwards, S. Liu, C.P. O’Brien, C.M. Gabardo, M. Fan, J.E. Huang, A. Robb, E.H. Sargent, A microchanneled solid electrolyte for carbon-efficient CO2 electrolysis, Joule, 6 (2022) 1333-1343.

[15] Y. Xie, P. Ou, X. Wang, Z. Xu, Y.C. Li, Z. Wang, J.E. Huang, J. Wicks, C. McCallum, N. Wang, High carbon utilization in CO_2_ reduction to multi-carbon products in acidic media, Nature Catalysis, 5 (2022) 564-570.

[16] C.P. O’Brien, R.K. Miao, S. Liu, Y. Xu, G. Lee, A. Robb, J.E. Huang, K. Xie, K. Bertens, C.M. Gabardo, Single pass CO_2_ conversion exceeding 85% in the electrosynthesis of multicarbon products via local CO_2_ regeneration, ACS Energy Letters, 6 (2021) 2952-2959.

[17] Y. Zhao, L. Hao, A. Ozden, S. Liu, R.K. Miao, P. Ou, T. Alkayyali, S. Zhang, J. Ning, Y. Liang, Conversion of CO_2_ to multicarbon products in strong acid by controlling the catalyst microenvironment, Nature Synthesis, 2 (2023) 403-412.

[18] A. Shayesteh Zeraati, F. Li, T. Alkayyali, R. Dorakhan, E. Shirzadi, F. Arabyarmohammadi, C.P. O’Brien, C.M. Gabardo, J. Kong, A. Ozden, M. Zargartalebi, Y. Zhao, L. Fan, P. Papangelakis, D. Kim, S. Park, R.K. Miao, J.P. Edwards, D. Young, A.H. Ip, E.H. Sargent, D. Sinton, Carbon- and energy-efficient ethanol electrosynthesis via interfacial cation enrichment, Nature Synthesis, (2024).

[19] M. Perfecto-Irigaray, J. Albo, G. Beobide, O. Castillo, A. Irabien, S. Pérez-Yáñez, Synthesis of heterometallic metal–organic frameworks and their performance as electrocatalyst for CO2 reduction, RSC Advances, 8 (2018) 21092-21099.

[20] B. Yang, L. Chen, S. Xue, H. Sun, K. Feng, Y. Chen, X. Zhang, L. Xiao, Y. Qin, J. Zhong, Z. Deng, Y. Jiao, Y. Peng, Electrocatalytic CO_2_ reduction to alcohols by modulating the molecular geometry and Cu coordination in bicentric copper complexes, Nature Communications, 13 (2022) 5122.

[21] C. Peng, G. Luo, J. Zhang, M. Chen, Z. Wang, T.-K. Sham, L. Zhang, Y. Li, G. Zheng, Double sulfur vacancies by lithium tuning enhance CO_2_ electroreduction to n-propanol, Nature Communications, 12 (2021) 1580.

[22] X. Su, Z. Jiang, J. Zhou, H. Liu, D. Zhou, H. Shang, X. Ni, Z. Peng, F. Yang, W. Chen, Complementary operando spectroscopy identification of in-situ generated metastable charge-asymmetry Cu_2_-CuN_3_ clusters for CO_2_ reduction to ethanol, Nature Communications, 13 (2022) 1322.

[23] X. Zhang, J. Li, Y.-Y. Li, Y. Jung, Y. Kuang, G. Zhu, Y. Liang, H. Dai, Selective and high current CO2 electro-reduction to multicarbon products in near-neutral KCl electrolytes, Journal of the American Chemical Society, 143 (2021) 3245-3255.

[24] G. Ma, O.A. Syzgantseva, Y. Huang, D. Stoian, J. Zhang, S. Yang, W. Luo, M. Jiang, S. Li, C. Chen, M.A. Syzgantseva, S. Yan, N. Chen, L. Peng, J. Li, B. Han, A hydrophobic Cu/Cu2O sheet catalyst for selective electroreduction of CO to ethanol, Nature Communications, 14 (2023) 501.

[25] T.T. Hoang, S. Verma, S. Ma, T.T. Fister, J. Timoshenko, A.I. Frenkel, P.J. Kenis, A.A. Gewirth, Nanoporous copper–silver alloys by additive-controlled electrodeposition for the selective electroreduction of CO_2_ to ethylene and ethanol, Journal of the American Chemical Society, 140 (2018) 5791-5797.

[26] L. Ding, N. Zhu, Y. Hu, Z. Chen, P. Song, T. Sheng, Z. Wu, Y. Xiong, Over 70 % Faradaic Efficiency for CO2 Electroreduction to Ethanol Enabled by Potassium Dopant-Tuned Interaction between Copper Sites and Intermediates, Angewandte Chemie International Edition, 61 (2022) e202209268.

[27] S. Kuang, Y. Su, M. Li, H. Liu, H. Chuai, X. Chen, E.J.M. Hensen, T.J. Meyer, S. Zhang, X. Ma, Asymmetrical electrohydrogenation of CO_2_ to ethanol with copper/gold heterojunctions, Proceedings of the National Academy of Sciences, 120 (2023) e2214175120.

[28] Y.C. Li, Z. Wang, T. Yuan, D.-H. Nam, M. Luo, J. Wicks, B. Chen, J. Li, F. Li, F.P.G. De Arquer, Binding site diversity promotes CO_2_ electroreduction to ethanol, Journal of the American Chemical Society, 141 (2019) 8584-8591.

[29] H. Wu, J. Li, K. Qi, Y. Zhang, E. Petit, W. Wang, V. Flaud, N. Onofrio, B. Rebiere, L. Huang, C. Salameh, L. Lajaunie, P. Miele, D. Voiry, Improved electrochemical conversion of CO_2_ to multicarbon products by using molecular doping, Nature Communications, 12 (2021) 7210.

[30] H. Wang, X. Bi, Y. Yan, Y. Zhao, Z. Yang, H. Ning, M. Wu, Efficient Electrocatalytic Reduction of CO_2_ to Ethanol Enhanced by Spacing Effect of Cu/Cu in Cu_2-x_Se Nanosheets, Advanced Functional Materials, 33 (2023) 2214946.

[31] W. Xia, Y. Xie, S. Jia, S. Han, R. Qi, T. Chen, X. Xing, T. Yao, D. Zhou, X. Dong, J. Zhai, J. Li, J. He, D. Jiang, Y. Yamauchi, M. He, H. Wu, B. Han, Adjacent Copper Single Atoms Promote C–C Coupling in Electrochemical CO_2_ Reduction for the Efficient Conversion of Ethanol, Journal of the American Chemical Society, 145 (2023) 17253-17264.

[32] X. Su, Y. Sun, L. Jin, L. Zhang, Y. Yang, P. Kerns, B. Liu, S. Li, J. He, Hierarchically porous Cu/Zn bimetallic catalysts for highly selective CO_2_ electroreduction to liquid C2 products, Applied Catalysis B: Environmental, 269 (2020) 118800.

[33] G. Wu, C. Zhu, J. Mao, G. Li, S. Li, X. Dong, A. Chen, Y. Song, G. Feng, X. Liu, Y. Wei, J. Wang, W. Wei, W. Chen, Ampere-Level CO_2_-to-Ethanol Conversion via Boron-Incorporated Copper Electrodes, ACS Energy Letters, 8 (2023) 4867-4874.

[34] Z.-Z. Wu, X.-L. Zhang, Z.-Z. Niu, F.-Y. Gao, P.-P. Yang, L.-P. Chi, L. Shi, W.-S. Wei, R. Liu, Z. Chen, S. Hu, X. Zheng, M.-R. Gao, Identification of Cu(100)/Cu(111) Interfaces as Superior Active Sites for CO Dimerization During CO_2_ Electroreduction, Journal of the American Chemical Society, 144 (2022) 259-269.

[35] X. Wang, Y. Jiang, K. Mao, W. Gong, D. Duan, J. Ma, Y. Zhong, J. Li, H. Liu, R. Long, Y. Xiong, Identifying an Interfacial Stabilizer for Regeneration-Free 300 h Electrochemical CO_2_ Reduction to C_2_ Products, Journal of the American Chemical Society, 144 (2022) 22759-22766.

[36] G. Zhang, Z.-J. Zhao, D. Cheng, H. Li, J. Yu, Q. Wang, H. Gao, J. Guo, H. Wang, G.A. Ozin, Efficient CO_2_ electroreduction on facet-selective copper films with high conversion rate, Nature communications, 12 (2021) 5745.

[37] C. Guo, Y. Guo, Y. Shi, X. Lan, Y. Wang, Y. Yu, B. Zhang, Electrocatalytic Reduction of CO2 to Ethanol at Close to Theoretical Potential via Engineering Abundant Electron-Donating Cu_δ+_ Species, Angewandte Chemie International Edition, 61 (2022) e202205909.

[38] W. Sun, P. Wang, Y. Jiang, Z. Jiang, R. Long, Z. Chen, P. Song, T. Sheng, Z. Wu, Y. Xiong, V-Doped Cu2Se Hierarchical Nanotubes Enabling Flow-Cell CO_2_ Electroreduction to Ethanol with High Efficiency and Selectivity, Advanced Materials, 34 (2022) 2207691.

[39] J. Li, R. Cai, H. Mu, J. Guo, X. Zhong, J. Wang, X. Du, J. Zhang, F. Li, Twin Heterostructure Engineering and Facet Effect Boosts Efficient Reduction CO_2_-to-Ethanol at Low Potential on Cu_2_O@Cu_2_S Catalysts, ACS Catalysis, 14 (2024) 3266-3277.

[40] M. Luo, Z. Wang, Y.C. Li, J. Li, F. Li, Y. Lum, D.-H. Nam, B. Chen, J. Wicks, A. Xu, T. Zhuang, W.R. Leow, X. Wang, C.-T. Dinh, Y. Wang, Y. Wang, D. Sinton, E.H. Sargent, Hydroxide promotes carbon dioxide electroreduction to ethanol on copper via tuning of adsorbed hydrogen, Nature Communications, 10 (2019) 5814.

[41] H. Xu, D. Rebollar, H. He, L. Chong, Y. Liu, C. Liu, C.-J. Sun, T. Li, J.V. Muntean, R.E. Winans, Highly selective electrocatalytic CO2 reduction to ethanol by metallic clusters dynamically formed from atomically dispersed copper, Nature Energy, 5 (2020) 623-632.

[42] K. Zhang, J. Wang, W. Zhang, H. Yin, J. Han, X. Yang, W. Fan, Y. Zhang, P. Zhang, Regulated Surface Electronic States of CuNi Nanoparticles through Metal-Support Interaction for Enhanced Electrocatalytic CO_2_ Reduction to Ethanol, Small, 19 (2023) 2300281.

[43] Y. Zang, T. Liu, P. Wei, H. Li, Q. Wang, G. Wang, X. Bao, Selective CO_2_ electroreduction to ethanol over a carbon‐coated CuOx catalyst, Angewandte Chemie International Edition, 61 (2022) e202209629.

[44] R. Purbia, S.Y. Choi, C.H. Woo, J. Jeon, C.W. Lim, D.K. Lee, J.Y. Choi, H.-S. Oh, J.M. Baik, Highly Selective and Low-overpotential Electrocatalytic CO_2_ Reduction to Ethanol by Cu-Single Atoms Decorated N-doped Carbon Dots, Applied Catalysis B: Environmental, (2024) 123694.

[45] F. Xu, B. Feng, Z. Shen, Y. Chen, L. Jiao, Y. Zhang, J. Tian, J. Zhang, X. Wang, L. Yang, Q. Wu, Z. Hu, Oxygen-Bridged Cu Binuclear Sites for Efficient Electrocatalytic CO_2_ Reduction to Ethanol at Ultralow Overpotential, Journal of the American Chemical Society, 146 (2024) 9365-9374.

[46] Y. Lin, T. Wang, L. Zhang, G. Zhang, L. Li, Q. Chang, Z. Pang, H. Gao, K. Huang, P. Zhang, Z.-J. Zhao, C. Pei, J. Gong, Tunable CO_2_ electroreduction to ethanol and ethylene with controllable interfacial wettability, Nature Communications, 14 (2023) 3575.

[47] D. Karapinar, N.T. Huan, N. Ranjbar Sahraie, J. Li, D. Wakerley, N. Touati, S. Zanna, D. Taverna, L.H. Galvão Tizei, A. Zitolo, F. Jaouen, V. Mougel, M. Fontecave, Electroreduction of CO_2_ on Single-Site Copper-Nitrogen-Doped Carbon Material: Selective Formation of Ethanol and Reversible Restructuration of the Metal Sites, Angewandte Chemie International Edition, 58 (2019) 15098-15103.

[48] Y. Liu, Y. Zhang, K. Cheng, X. Quan, X. Fan, Y. Su, S. Chen, H. Zhao, Y. Zhang, H. Yu, Selective electrochemical reduction of carbon dioxide to ethanol on a boron‐and nitrogen‐Co‐doped nanodiamond, Angewandte Chemie, 129 (2017) 15813-15817.

[49] Y. Song, S. Wang, W. Chen, S. Li, G. Feng, W. Wei, Y. Sun, Enhanced Ethanol Production from CO_2_ Electroreduction at Micropores in Nitrogen-Doped Mesoporous Carbon, ChemSusChem, 13 (2020) 293-297.

[50] J. Ding, H. Bin Yang, X.-L. Ma, S. Liu, W. Liu, Q. Mao, Y. Huang, J. Li, T. Zhang, B. Liu, A tin-based tandem electrocatalyst for CO_2_ reduction to ethanol with 80% selectivity, Nature Energy, 8 (2023) 1386-1394.
